# Tumor and local lymphoid tissue interaction determines prognosis in high-grade serous ovarian cancer

**DOI:** 10.1016/j.xcrm.2023.101092

**Published:** 2023-06-21

**Authors:** Haonan Lu, Hantao Lou, Georg Wengert, Reema Paudel, Naina Patel, Saral Desai, Bill Crum, Kristofer Linton-Reid, Mitchell Chen, Dongyang Li, Jacey Ip, Francesco Mauri, David J. Pinato, Andrea Rockall, Susan J. Copley, Sadaf Ghaem-Maghami, Eric O. Aboagye

**Affiliations:** 1 Department of Surgery and Cancer, Imperial College, Hammersmith Campus, The Commonwealth Building, Du Cane Road, W12 0NN London, UK; 2 Ludwig Cancer Research, Nuffield Department of Medicine, University of Oxford, OX3 7DQ Oxford, UK; 3 Imperial College Healthcare NHS Trust, Du Cane Road, W12 0HS London, UK; 4 Division of Oncology, Department of Translational Medicine, University of Piemonte Orientale, Novara, Italy

**Keywords:** ovarian cancer, tertiary lymphoid structures, CNA, radiomics

## Abstract

Tertiary lymphoid structure (TLS) is associated with prognosis in copy-number-driven tumors, including high-grade serous ovarian cancer (HGSOC), although the function of TLS and its interaction with copy-number alterations in HGSOC are not fully understood. In the current study, we confirm that TLS-high HGSOC patients show significantly better progression-free survival (PFS). We show that the presence of TLS in HGSOC tumors is associated with B cell maturation and cytotoxic tumor-specific T cell activation and proliferation. In addition, the copy-number loss of *IL15* and *CXCL10* may limit TLS formation in HGSOC; a list of genes that may dysregulate TLS function is also proposed. Last, a radiomics-based signature is developed to predict the presence of TLS, which independently predicts PFS in both HGSOC patients and immune checkpoint inhibitor (ICI)-treated non-small cell lung cancer (NSCLC) patients. Overall, we reveal that TLS coordinates intratumoral B cell and T cell response to HGSOC tumor, while the cancer genome evolves to counteract TLS formation and function.

## Highlights

• Tertiary lymphoid structure, or TLS, predicts survival in ovarian cancer
• TLS is associated with both B cell and T cell activation
• Chromosome 4q deletion/DCAF15 amplification are linked to TLS loss/dysfunction
• A radiomics-based predictor of TLS is developed

## Graphical abstract

**Figure:**
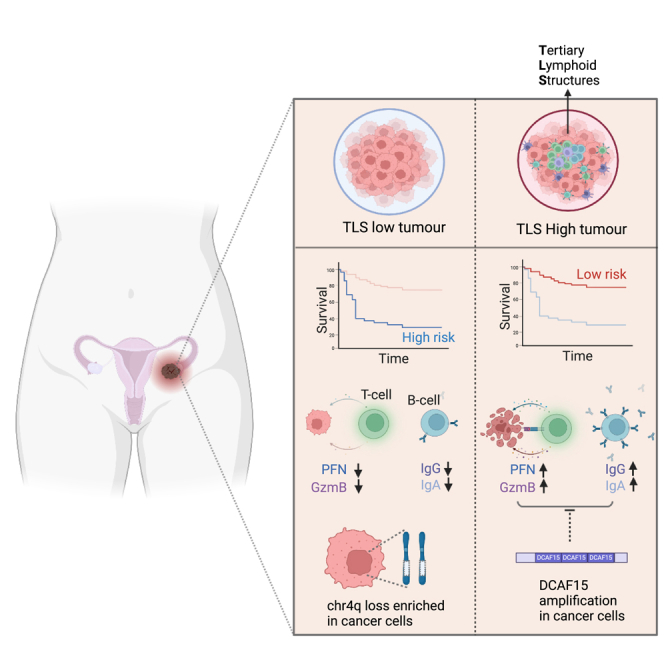

## Teaser

Lu et al. present the immunogenomic analysis of 242 high-grade serous ovarian cancer cases. They confirm the clinical and biological significance of tertiary lymphoid structures and identify chr4q loss and DCAF15 amplification, which could influence the formation and functions of tertiary lymphoid structures.

## Introduction

Clinically available immune checkpoint inhibitors (ICI) restore patients’ T cell functions to recognize and clear cancer cells. ICIs are effective for a large proportion of oncological indications, including non-small cell lung cancer (NSCLC).^1^ Nevertheless, a few cancer types, including high-grade serous ovarian cancer (HGSOC), respond very poorly to T cell-based immunotherapy (e.g., anti-PD1 and anti-PD-L1 agents).^2^^,^^3^^,^^4^ One obvious explanation for the lack of response is that HGSOC is not typically driven by point mutations.^5^^,^^6^ It has a very low tumor mutational burden (TMB) and therefore is characterized by a lower tumor neoantigen diversity, leading to lower intrinsic immunogenicity.^5^ The low efficacy of ICI is in deep contrast to accumulating evidence showing high prognostic importance of the immune cell infiltrate in HGSOC. A number of studies confirmed that HGSOC patients with increased intraepithelial CD8^+^ tumor-infiltrating lymphocytes (TILs) had significantly better overall survival^7^^,^^8^^,^^9^; neoepitope-specific T cells were identified in TILs and the neoantigens were capable of evoking an IFNγ response, as well as TNF-α and IL-2 secretion.^10^ Understanding the mechanisms of therapeutic vulnerability within the HGSOC tumor microenvironment is an area with a high unmet need.

Tertiary lymphoid structure (TLS) is an ectopic lymphoid organ that is observed at the inflammation site in some autoimmune diseases and cancer.^11^ The TLS comprises mainly B cells, T cells, and myeloid dendritic cells and is often formed in the stroma upon chronic inflammatory stimulation via cytokines and chemokines.^11^ Secondary lymphoid organs (SLOs) and TLSs share many similarities, although differences exist: (1) formation of SLOs, including spleen, lymph nodes, and Peyer’s patch, occurs during embryonic life and is part of the normal development process, whereas TLSs are formed in organs of chronic inflammation and are part of a pathogenic process.^12^ (2) SLOs are often highly structured, whereas TLSs can have heterogeneous shape, size, and function.^13^ (3) SLOs display complex anatomical compartmentalization, whereas TLSs do not.^12^ TLSs require a persistent expression of lymphoid chemokines, including CXCL13, CCL19, and CCL21, by stromal cells to attract CXCR4/CXCR5-expressing B cells and CCR7-expressing T cells to form the B cell zone and T cell zone, respectively.^14^ Naive B cells are attracted to the CXCL13 gradient, entering the TLS to initiate the classical germinal center reaction; T cells are also attracted and polarized by cytokines including IL-12 and IL-4 within the TLS. Recent studies showed that the TLS is often enriched in tumors of lung cancer and melanoma patients who respond better to ICIs.^15^^,^^16^^,^^17^ Mechanistically, B cells in the TLS were proposed to facilitate T cell activation, and T cells from TLS-low melanoma tumors displayed a dysfunctional phenotype.^16^ Furthermore, B cells were previously found to be associated with prognosis in HGSOC,^18^^,^^19^^,^^20^ and immunoglobulins produced by B cells delivered an anti-tumor response.^21^^,^^22^ TLS was also recently found to be associated with prognosis in HGSOC.^20^^,^^23^ Nevertheless, the complete function of the TLS is to be generalized in a representative HGSOC cohort.

Cancer cells evolve stepwise to acquire cancer hallmarks, including immune evasion.^24^ Point mutation and copy-number alteration (CNA) are the two most common mechanisms to facilitate this process.^6^ For example, *KEAP1* mutations in predominantly mutation-driven cancers (e.g., NSCLC) were found to drive T cell exclusion and immunotherapy resistance, partly via metabolic reprogramming and type I interferon suppression.^25^^,^^26^^,^^27^ For CNA-driven tumors, e.g., HGSOC, often a set of genes is amplified or deleted to acquire the hallmarks. A recent meta-analysis revealed that 9q34 loss and *CCND1* amplification are associated with ICI treatment resistance^28^; *PTEN* loss was associated with a strong immunosuppressive microenvironment mediated by myeloid-derived suppressor cells, regulatory cells, and M2 macrophages.^29^ However, little is known regarding how the global CNA profile could interact with the anti-tumor immunity in those CNA-driven tumors.

Radiomics is a quantitative imaging analysis of standard-of-care medical images. It summarizes imaging features including shape and size, texture, and wavelet decomposition, which contain biological and clinically relevant information about the tumor. Given its non-invasive and cost-effective nature, interest in the development of radiomics as a predictive and prognostic biomarker is growing. We previously developed a radiomics-based prognostic vector (RPV) for HGSOC, which predicts HGSOC patient survival, and recently validated the RPV in a large independent validation cohort.^30^^,^^31^ We also showed that the RPV was strongly linked to the proportion of reactive stroma, which confers resistance to treatment in HGSOC.^30^ A number of studies also showed that some radiomics features could predict T cell infiltration in lung, gastric, and liver cancers.^32^^,^^33^^,^^34^ A recent study developed a radiomics-based biomarker that successfully predicted CD8 T cell infiltration and response to immunotherapy in a clinical trial cohort containing a range of solid tumors.^35^ Since TLS is a structural feature of the microenvironment, we hypothesized that radiomics may predict the presence of TLS in tumors and therefore could be used as a candidate biomarker for immunotherapy.

In this study, we aim to confirm the function of the TLS in conferring local anti-tumor immunity as well as to understand how different CNAs affect TLS formation and function in HGSOC. We compared the findings with the immunogenic NSCLC cohort as a positive control. We additionally explored the use of radiomics as a biomarker of TLS to assist future clinical application.

## Results

### Prognostic impact of tertiary lymphoid structure

**Figure 1:**
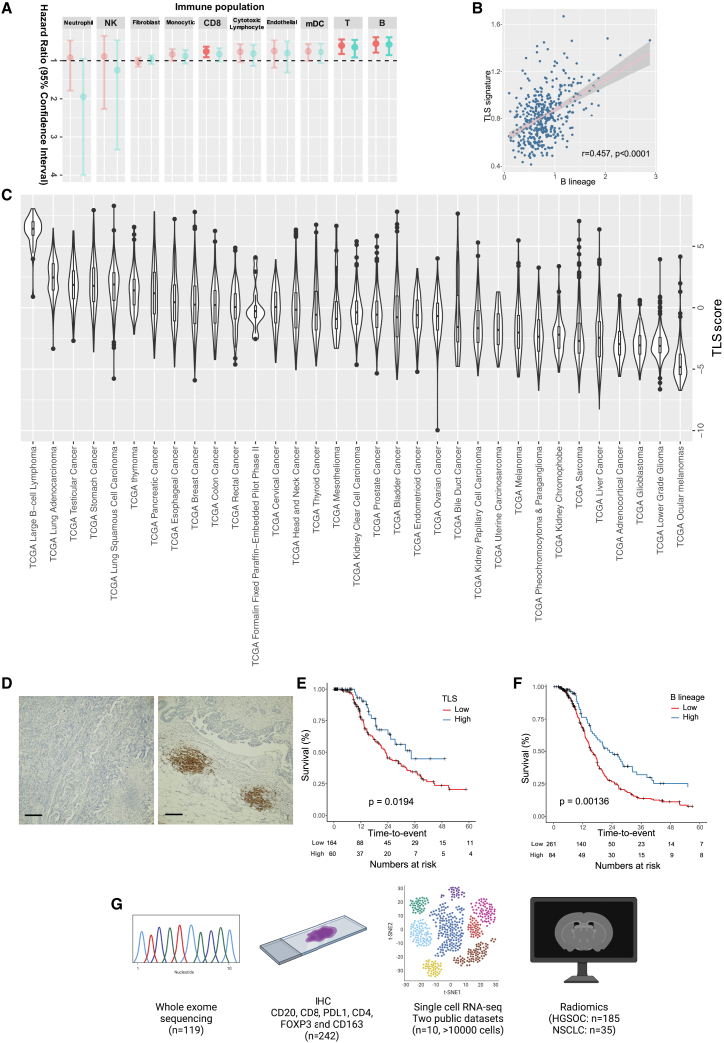
Prognostic impact of tertiary lymphoid structures in ovarian cancer (A) Summary of prognostic potential of immune cell subpopulations in TCGA ovarian cancer cohort. Hazard ratio of progression-free survival is plotted on the x axis, in a univariate Cox regression model (light blue) and multivariable Cox regression model (red); n = 345. Statistically significant association is shown in darker colors. (B) Correlation between B cells and TLS markers in TCGA cohort. (C) TLS abundance across cancer types in TCGA cohort. (D) Immunohistochemistry staining of CD20 in TLS-low (left) and TLS-high (right) ovarian tumor tissues. Scale bar indicates 200 μm. (E and F) Kaplan-Meier plots of TLS and progression-free survival in (E) HH cohort and (F) TCGA cohort. (G) Overview of the study. Whole-exome sequencing to define mutational and copy-number profiles; immunohistochemistry for immune markers and radiomics profile are collected from HH cohort; two public single-cell RNA-sequencing datasets are reanalyzed. The TLS signature is derived from the B lineage from MCPcounter.

Proportions of certain immune cell populations, including cytotoxic T cells, macrophages, and B cells, have been associated with patient prognosis in ovarian cancer.^8^^,^^36^^,^^37^ To systematically compare their prognostic impact, we first estimated intratumoral immune cell populations, including T cells, B cells, myeloid dendritic cells, monocytic cells, natural killer (NK) cells, and neutrophils, using MCPcounter^38^ and associated them with progression-free survival (PFS) in TCGA HGSOC and lung adenocarcinoma (LUAD) cohorts (Figures 1A and S1A). In both HGSOC and LUAD cohorts analyzed, B cells, T cells, and myeloid dendritic cells remained the top three populations associated with PFS. Of note, B cell and myeloid dendritic cell populations were significantly higher in LUAD compared with HGSOC (Figure S1B).

B cells, T cells, and myeloid dendritic cells are the three main components of TLS in cancer. We therefore quantified TLS using a previously described gene expression signature^16^ and found that the presence of TLS was significantly correlated with B cells (r = 0.457, p < 0.0001; Figure 1B), T cells (r = 0.493, p < 0.0001), and myeloid dendritic cells (r = 0.399, p < 0.0001) in the TCGA cohort. In addition, we quantified the presence of TLS in 33 human cancers using TCGA cohorts and confirmed that HGSOC had a relatively lower TLS content compared with other tumors, including lung and esophageal cancers (Figure 1C).

To validate the prognostic value of TLS in HGSOC, we performed immunohistochemistry of CD20 (B cell surface marker) on 242 HGSOC tumor sections from Hammersmith Hospital, London (HH cohort; Figures 1D and S1C and Table 1). The biospecimens included in the HH cohort were all from primary ovarian tumors of treatment-naive patients. The patients from the TLS-high and TLS-low groups showed similar characteristics such as age at diagnosis, FIGO stage, post-surgical residual disease, and post-surgical treatment (p > 0.05; Table 1). The CD20 staining in the primary ovarian tumor tissues co-localized with CD4^+^, CD8^+^ T cells and Ki67, suggesting these were true lymphoid structures, and tumor content was not associated with TLS status, suggesting that the presence of TLS was unlikely to be the result of sampling bias (Figures S1C–S1E). Kaplan-Meier analysis of the HH cohort confirmed that HGSOC patients with higher TLS score had better PFS (Figure 1E), which is consistent with the TCGA HGSOC cohort (Figure 1F). Moreover, the association between TLS and PFS was independent of known clinical prognosticators, including stage, age, and residual disease, from multivariable Cox regression analysis (hazard ratio [HR] = 0.55, 95% CI 0.44–0.94, p = 0.0292). In addition to the immunohistochemistry for immune markers, we integrated two public single-cell RNA-sequencing datasets to elucidate the immunological phenotypes of TLS in HGSOC; we also collected radiomics profiles for both HGSOC and NSCLC patients in the HH cohorts to develop a predictive biomarker of TLS (Figure 1G).

**Table 1:** Clinical characteristics of HGSOC cases from the HH cohort

Characteristic	TLS low (n = 179)	TLS high (n = 63)	p
Age, years (%)			
<70	135 (75.4)	46 (73)	ns
>70	44 (24.6)	17 (27)	
FIGO stage (%)			
I	9 (5)	2 (3.2)	ns
II	12 (6.7)	7 (11.1)	
III	107 (60)	38 (60.3)	
IV	45 (25.1)	15 (23.9)	
unknown	6 (3.3)	1 (1.6)	
Residual disease (%)			
optimal	128(71.5)	51(81)	ns
suboptimal	40(22.3)	9(14.3)	
unknown	11 (6.1)	3 (4.8)	
PFS (months)			
median	23	34.4	0.0194
IQR	13–44.8	18.6–∗	
Relapse (%)			
no	79(44.1)	44(69.8)	0.00151
yes	85(47.5)	17(27)	
unknown	15(8.4)	2 (3.2)	

The results here suggest that the presence of TLS may have a strong impact on suppressing tumor progression in HGSOC, while the biological function of TLS in HGSOC remains elusive.

### Function of B cells from TLS

**Figure 2:**
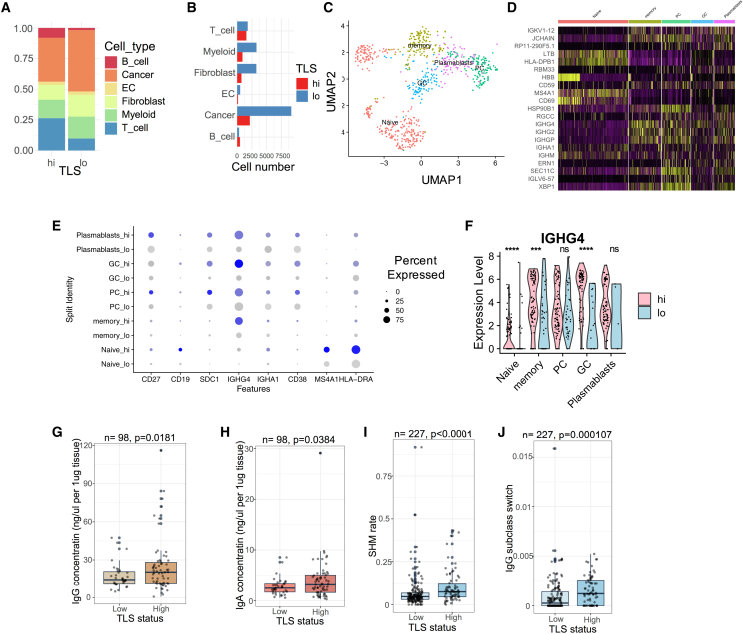
Function of B cells within tertiary lymphoid structures in ovarian cancer (A and B) (A) Proportions and (B) cell numbers of cell subtypes in TLS-low (n = 3; 18,354 cells) ovarian tumors compared with TLS-high (n = 2; 5,996 cells) ovarian tumors. (C) UMAP showing five B cell subtypes in ovarian tumors. Cell cluster annotation for (C–F): naive cells, memory cells, plasma cells (PCs), germinal center (GC), and plasmablasts. (D) Marker genes in the five B cell subtypes. Yellow indicates high expression and purple indicates low expression. (E) Dot plot showing marker genes in five B cell subtypes comparing TLS-high with TLS-low tumors. Blue represents cells from the TLS-high group, gray represents cells from the TLS-low group. (F) *IGHG4* expression across B cell subtypes. The p-values are given by Wilcoxon rank-sum test. ∗∗∗∗p < 0.0001; ∗∗∗p < 0.001; ns, p > 0.05. (G and H) Concentrations of ovarian tumor-derived (G) IgG and (H) IgA comparing TLS-high (n = 63) with TLS-low tumors (n = 35) from the HH cohort. (I and J) (I) Somatic hypermutation of CDR3 sequences and (J) IgG3-to-IgG1 subclass switching rate comparing TLS-high (n = 57) with TLS-low tumors (n = 170) in the TCGA ovarian cancer cohort. For (G)–(J), the p values are given by two-tailed t test. For boxplot, elements are defined as follows: the center line indicates median value, box limits indicate upper and lower quartiles, whiskers extend to 1.5× the interquartile range, and points beyond the whiskers are outliers.

To understand the biological importance of TLSs in HGSOC, we first reanalyzed a single-cell RNA-sequencing dataset containing a total of 24,350 single cells from five HGSOC tumors.^39^ The entire cell population from the five tumors was clustered, and cell types were identified (B cells, T cells, myeloid cells, endothelial cells, and fibroblasts) based on marker expression (Figure S2). We next split the five tumors into TLS high (n = 2; 5,996 cells) and TLS low (n = 3; 18,354 cells) based on the B cell content in each tumor (Figure 2A). By examining the proportion of each cell population, TLS-high tumors contain more T cells but fewer cancer cells compared with TLS-low tumors (Figure 2A). To ensure that the downstream analysis is independent of number of cells sequenced, the cell number in each population was also compared between TLS-high and TLS-low tumors (Figure 2B). The numbers of B cells and T cells sequenced from the two groups were similar.

To understand the differences in B cell populations from TLS, we subgrouped B cells into four cell types and visualized them using uniform manifold approximation and projection (UMAP) (Figures 2C, S2C, and S2D). By considering the marker genes expressed in each cell cluster, we assigned them as plasma cell (SDC1^+^), naive cell (MS4A1^+^), plasmablast (CD27^+^), memory cell (IGHG4^+^ and SDC1^−^), and germinal center (CD38^+^) B cells. In all the five B cell subgroups, we observed stronger expression of immunoglobulin family genes, including IGHA1 and IGHG4, in TLS-high tumors compared with TLS-low tumors (Figures 2D–2F). A similar finding was seen in two independent single-cell RNA-sequencing datasets (Figure S3). Next, we extracted and quantified polyclonal IgA and IgG antibodies from 98 HGSOC tumor tissues from the HH cohort. Consistent with the *in silico* results (Figures 2E, S3F, and S3L), IgA and IgG antibody concentrations were significantly higher in TLS-high tumors (Figures 2G and 2H). The results suggest that TLS enhances the local production of antibodies within tumors.

To assess the tumor specificity of TLS-derived antibodies, we first tested the binding of polyclonal IgG antibodies with cancer cells. Using immunofluorescence microscopy, we observed specific binding of polyclonal antibodies extracted from two tumor tissues to HGSOC cell lines (OVCAR3 and OVCAR4), while the binding was not observed in a non-malignant cell line (OSEC) (Figure S4A and S4B).

Next, we inferred the B cell maturation process using the TRUST algorithm and RNA-sequencing data.^40^ In the TCGA cohort, both somatic hypermutation rate and IgG3-to-IgG1 subclass switch rate were significantly elevated in ovarian tumors with higher TLS (Figures 2J and 2K), implying that TLS may facilitate a localized B cell maturation process against HGSOC tumors.

Collectively, these results suggest that TLS is associated with tumor-specific B cell maturation and antibody production, and thus, potentially contributing to the local immunity against ovarian tumor.

### Function of T cells from TLS

**Figure 3:**
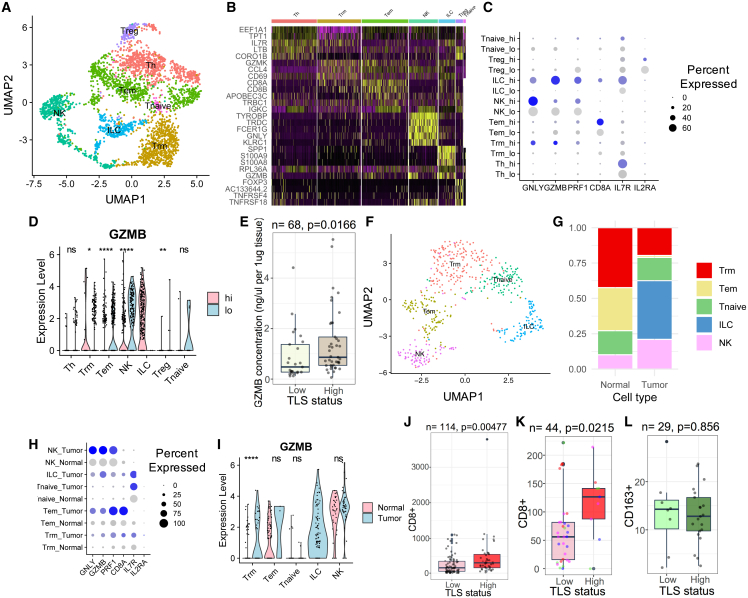
Function of T cells within tertiary lymphoid structures in ovarian cancer (A) UMAP showing seven T cell subpopulations in ovarian tumors. Cell cluster annotation for (A–D): CD4^+^ T helper cells (Th), CD8^+^ T resident memory (Trm), effector memory T cells (Tem), NK cells (NK), CD4^+^ GZMB^+^ (ILC), regulatory T cells (Treg), and CD4^+^ IL-7R^+^ naive T cells (Tnaive). (B) Marker genes in the seven T cell subpopulations. (C) Dot plot showing marker genes in T cell subpopulations comparing TLS-high with TLS-low tumors. (D) *GZMB* expression in T cell subpopulations comparing TLS-high with TLS-low tumors. The p-values are given by Wilcoxon rank-sum test. (E) GZMB concentration comparing TLS-high (n = 44) with TLS-low tumors (n = 24) from HH cohort. (F) UMAP showing five T cell subpopulations in ovarian tumors and adjacent non-malignant tissues. Cell cluster annotation for (F–I): CD8^+^ T resident memory (Trm), effector memory T cells (Tem), NK cells (NK), CD4^+^ GZMB^+^ (ILC), and CD4^+^ IL-7R^+^ naive T cells (Tnaive). (G) Proportions of T cell subpopulations comparing ovarian tumors with adjacent non-malignant tissues. (H) Dot plot showing marker gene expression in tumor and non-malignant tissues. (I) *GZMB* expression in T cell subpopulations comparing tumor with non-malignant tissues. The p-values are given by Wilcoxon rank-sum test. (J) Numbers of CD8^+^ T cells that infiltrated TLS-high ovarian tumors (n = 41) compared with TLS-low ovarian tumors (n = 73) in the HH cohort. Non-TLS CD8^+^ T cells were measured. (K) Numbers of CD8^+^ T cells in TLS-high (n = 10) sections compared with paired TLS-low (n = 34) sections from the same ovarian cancer cases. (L) Numbers of CD163^+^ cells in TLS-high tumors (n = 21) compared with TLS-low tumors (n = 8) in the HH cohort. For (J)–(L), the p values are given by two-tailed t test. ∗∗∗∗p < 0.0001; ∗∗p < 0.01; ∗p < 0.05; ns, p > 0.05. For boxplot elements are defined as follows: the center line indicates median value, box limits indicate upper and lower quartiles, whiskers extend to 1.5× the interquartile range, and points beyond the whiskers are outliers.

Previous studies showed that CD8^+^ T cells were associated with better prognosis in HGSOC,^8^^,^^9^ prompting the question of whether TLS could promote the cytotoxic activity of T cells. We, therefore, used the same single-cell RNA-sequencing cohort and subgrouped the T cell population. First, we observed that a significantly higher proportion of T cells were present in the TLS-high tumors (Figures 2A and S2F–S2H). Consistent with the previous study,^41^ we observed seven T cell subtypes, namely CD4^+^ T helper cells (Th), CD8^+^ T resident memory cells (Trm), T effector memory cells (Tem), NK cells, CD4^+^ GZMB^+^ cells (ILC), regulatory T cells (Treg), and CD4^+^ IL-7R^+^ naive T cells (Tnaive) (Figure 3A). Among the seven T cell subtypes, we observed that the CD4^+^ GZMB^+^ ILC population, which exhibited the most cytotoxic phenotype (GNLY^+^, GZMB^+^, PRF1^+^, FCER1G and CD8A^+^), was present exclusively in the TLS-high tumors (Figures 3B–3D). This cell population was also found to be enriched in the TLS-high tumors in two independent single-cell RNA-sequencing datasets (Figures S3D and S3J). This unique CD4^+^ GZMB^+^ ILC population shared great similarity with recently discovered intratumoral innate-like T cells with high cytotoxic potential (ILTCKs), which showed phenotypes of both T cells (CD8^+^ and TCRβ^+^) and NK cells (FCER1G^+^ and GZMB^+^).^42^^,^^43^ Consistent with this finding, ELISA data using tumor tissues from the HH cohort indicated that TLS-high tumors expressed significantly higher amounts of GZMB compared with TLS-low tumors (Figure 3E). The results suggest that cytotoxic T cell populations from TLS-high tumors are more activated compared with TLS-low tumors.

A number of studies reported the presence of bystander T cells in tumors, which are not tumor specific.^44^^,^^45^ We therefore analyzed T cells in HGSOC tumors compared with the same population in adjacent normal tissues in an independent single-cell RNA-sequencing dataset (n = 4, 5,649 cells).^46^ Through re-subgrouping of the T cell population, we observed Trm, Tem, Tnaive, ILC, and NK cells in both normal and tumor tissues (Figure 3F). We consistently observed the strong cytotoxic CD4^+^ GZMB^+^ ILC population enriched in HGSOC tumors compared with normal tissues (Figures 3G–3I), implying that the cytotoxic T cell populations were specific against HGSOC tumors. The Th and Treg populations were missing in this cohort, possibly due to insufficient T cells sequenced.

Since T cells that have infiltrated HGSOC tumors could originate from either TLS or SLOs, we hypothesized that TLS could enhance the local proliferation and infiltration of T cells into tumors. We, therefore, performed immunohistochemistry (IHC) to quantify the presence of CD8^+^ T cells on 114 HGSOC tumor tissues from the HH cohort. Consistent with single-cell RNA-sequencing data, we found that CD8^+^ T cells that were outside of the TLS were significantly enriched in TLS-high tumors compared with TLS-low tumors (Figures 3J and S1D). A similar observation was made in the TCGA HGSOC cohort, where tumors with higher TLS score were more enriched for T cells (p < 0.0001, unpaired t test; Figure S4C). T cell infiltration can be affected by multiple factors, including tissue stiffness and cancer cell signaling. To exclude non-TLS variables, we stained multiple tumor tissues from the same HGSOC patients (n = 11). We found that TLS distribution could be heterogeneous: from tumors with few TLSs present in 15/15 tissue sections to tumors with TLSs present in almost half (3/7) of the tissue sections. Since each group of tissue sections was from the same patient, they would be expected to have largely similar cancer cell phenotypes and tissue microenvironment properties. Interestingly, we consistently observed higher amounts of CD8^+^ T cells in TLS-high tissue sections compared with TLS-low tissue sections from the same patient (p = 0.0215, paired two-tailed t test; Figure 3K). Furthermore, we found that immune cells from the myeloid lineage were not associated with the presence of T cells from the HH cohort (Figure 3L), suggesting that TLS may enhance lymphoid lineage activation and proliferation specifically.

### Impact of copy-number alterations on tertiary lymphoid structure formation

While TLS in HGSOC exhibited anti-tumor phenotypes, HGSOC tumors had significantly less TLS content compared with LUAD, and the presence of TLS in individual HGSOC patients was extremely heterogeneous. Since HGSOC is heavily driven by CNAs, including deletion of tumor-suppressor genes,^5^ we hypothesized that genes involved in immune cell communication could be genetically altered in HGSOC cells to dysregulate TLS formation.

**Figure 4:**
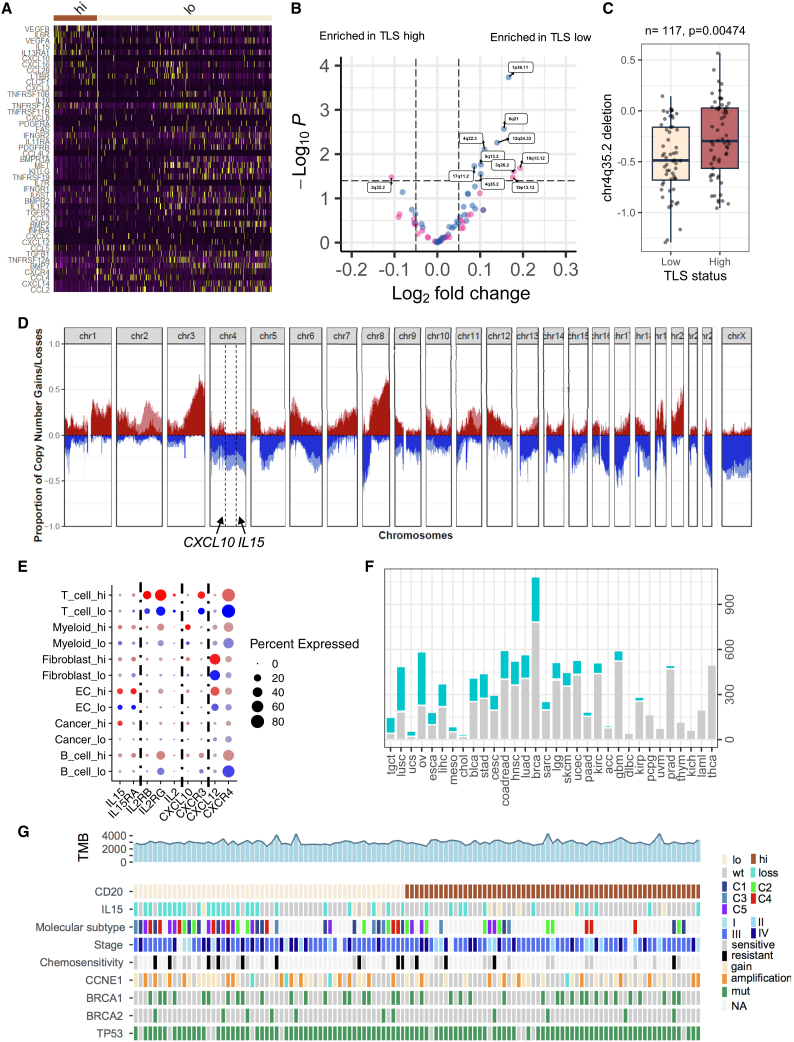
Copy-number alterations dysregulate tertiary lymphoid structure formation (A) Heatmap showing differentially expressed genes involved in cytokine signaling comparing cancer cells from TLS-high tumors with those from TLS-low tumors. (B) Volcano plot showing genomic gains and losses associated with TLSs from the TCGA cohort. Significantly enriched genomic regions are labeled (FDR < 0.25). Horizontal line indicates FDR = 0.25. The p-values are given by a moderated t test. Pink, copy number gains; blue, copy number deletions. TLS signature was derived from the B lineage from MCPcounter. (C) Boxplot showing association between chr4q35.2 and TLS status from the HH cohort. The p-value is given by two-tailed t test. TLS low, n = 56; TLS high, n = 61. (D) Proportion of copy-number alterations comparing TLS-high tumors (dark red and dark blue) with TLS-low tumors (light red and light blue) in the HH cohort. The genomic locations of *CXCL10* and *IL15* are indicated; x axis, chromosome number; y axis, proportion of copy-number gain (red, 0 to 1) and loss (blue, 0 to −1). (E) Dot plot showing cytokines and their receptor expression in cell subtypes. Dot color: red, cells in the TLS-high group; blue, cells in the TLS-low group. Dot size represents percentage of cells expressing the gene. (F) Number of tumors containing *IL15* deletion across cancer types; x axis, number of cases; cyan, cases with partial *IL15* deletion; gray, cases without *IL15* loss. (G) Heatmap showing *IL15* deletion and clinical phenotypes associated with TLS in the HH cohort. TMB, tumor mutational burden.

We first compared genes that were differentially expressed between TLS-high and TLS-low tumors within cancer cell populations from the single-cell RNA-sequencing dataset and specifically analyzed genes involved in cytokine-receptor interaction pathways from the KEGG database (Figure 4A). We found 10 genes, including *VEGFB*, *IL6R*, *VEGFA*, *IL15*, *IL13RA1*, and *CXCL10*, significantly downregulated in TLS-low tumors and 35 genes, including *CXCL3*, *TNFRSF10B*, *IL10*, *TNFRSF1A*, *TNFRSF11B*, and *CXCL8*, significantly upregulated in TLS-low tumors (Figure 4A).

The presence of TLS may have a significant impact on the transcriptional profile of cancer cells. Dysregulated genes with genetic mechanism such as CNAs may imply their cancer-driving role.^47^^,^^48^ By performing whole-exome sequencing of 117 HGSOC tumors, we associated genome-wide CNA events with TLS in the HH cohort and validated this finding using the TCGA HGSOC cohort (n = 407) (Figures 4B–4D). We found that the genomic region (chr4q35.2) containing *IL15* and *CXCL10*, which were downregulated in TLS-low tumors, was consistently lost in TLS-low tumors in both the TCGA (Figures 4B, S5A, and S5C;Table S1) and the HH cohorts (Figures 4C and 4D). In addition, the downregulation of IL-15 and CXCL10 in cancer cells from TLS-low tumors was also observed in an independent single-cell RNA-sequencing dataset (Figure S5E).

The loss of *IL15* and *CXCL10* also correlated with the loss of corresponding gene expression in the TCGA cohort, suggesting the functional impact of copy-number deletions of these two genes (Figures S5F and S5G). *IL15* and *CXCL10* were expressed in a subset of cancer cells with high TLS and the most macrophage populations^49^ (Figures 4E and S5E). To verify if the copy-number loss was an independent mechanism contributing to gene expression downregulation, we performed linear regression to associate both macrophage quantity and copy-number loss with its gene expression in the TCGA cohort. Both *IL15* and *CXCL10* copy-number loss was significantly associated with their corresponding gene expression in the multiple variable linear regression model (*IL15*, beta = 0.51, p = 9.32 × 10^−10^; *CXCL10*, beta = 0.76, p = 2.45 × 10^−6^), suggesting both copy number and macrophage infiltration as independent mechanisms for *IL15* and *CXCL10* expression in HGSOC tumors.

Strikingly, the associations of *IL15* and *CXCL10* with TLS were also found significant in both LUAD and lung squamous carcinoma patients (Figures S5B and S5D). Furthermore, over 50% of HGSOC patients and 31.2% of LUAD patients from the TCGA cohorts presented with *IL15* partial deletion (Figure 4F). In addition to CNAs, we also observed a significant association between *FMR1* mutation or *KEAP1* mutation and TLS loss in LUAD patients from the TCGA cohort, suggesting both CNA and mutation as potential mechanisms to limit TLS formation in lung tumors (Figures S5H and S5I).

Collectively, these results implied that copy-number deletions of essential cytokines and chemokines could be a general mechanism to disrupt TLS formation in human cancers.

### TLS and molecular phenotypes

We next investigated the potential association between TLS and known clinical and molecular prognosticators in HGSOC.

The association between TLS and age at diagnosis was found statistically significant: patients with TLS-low tumors were moderately younger compared with TLS-high patients. FIGO stage, post-operative residual disease, or primary chemotherapy response was not associated with TLS.

Molecular subtypes including mesenchymal (C1), immunoreactive (C2), differentiated (C4), and proliferative (C5), based on gene expression profile, were previously described in HGSOC.^5^^,^^50^ It was later found that the presence of different stromal cells significantly contributes to these molecular subtypes.^41^^,^^51^ Consistent with previous findings, we found that TLS was significantly enriched in the immunoreactive (C2) subtype of HGSOC (Figures 4G, S5J, and S5K).

Other known genetic alterations, including TMB, *BRCA1/2* mutations, *CCNE1* amplification, or *PTEN* deletion, were not found to be associated with TLS in either the HH or the TCGA cohort (Figure 4G).

### Impact of copy-number alterations on tertiary lymphoid structure function

**Figure 5:**
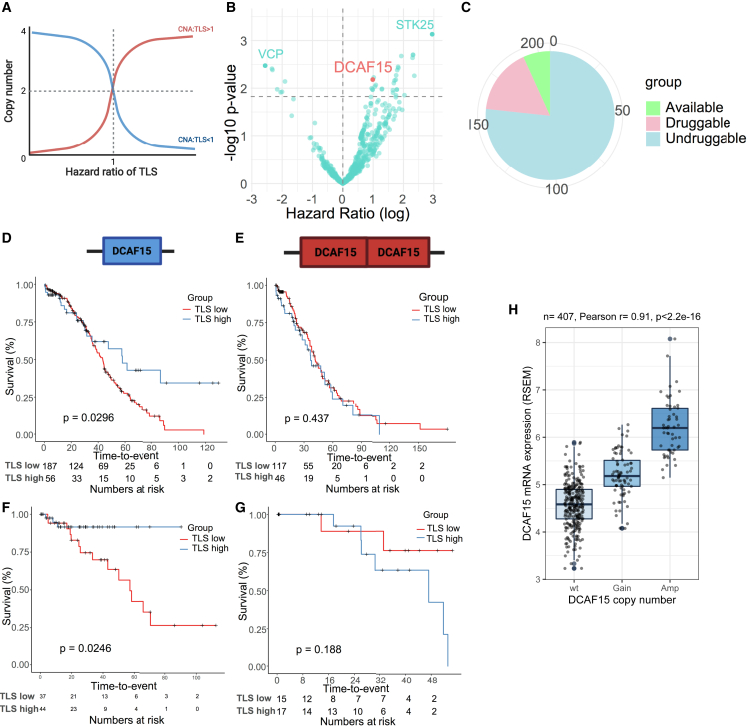
Identification of TLS-interacting CNA targets in ovarian cancer (TICTOC) (A) The association between CNA and hazard ratio of TLS when can/TLS interaction is greater than 1 (red) or less than 1 (blue). (B) Volcano plot showing CNAs that are statistically interacting with TLS in the multivariable Cox regression model for overall survival in the TCGA cohort. (C) Potential druggability of TICTOC targets. (D–G) Kaplan-Meier plots showing association between TLS and overall survival when DCAF15 is not amplified in (D) the TCGA cohort and (F) the HH cohort and when DCAF15 is amplified in (E) the TCGA cohort and (G) the HH cohort. The p-values are given by log rank test. (H) Correlation between DCAF15 expression and its copy-number alterations in the TCGA ovarian cancer cohort. Boxplot elements are defined as follows: the center line indicates median value, box limits indicate upper and lower quartiles, whiskers extend to 1.5× the interquartile range, and points beyond the whiskers are outliers.

Beyond affecting TLS formation, we hypothesized that some CNAs may evolve to counteract TLS functions to evade local immunity. To discover these potential TLS-interacting genes, we first performed multivariable Cox regression analysis including TLS, individual CNA, and TLS/CNA interactions as covariates and overall survival as outcome in the TCGA cohort (Figure 5A and Table S2). From the preliminary analysis, 356 copy-number gains (HR > 1 and false discovery rate [FDR] < 25%; e.g., STK25 and DCAF15) and 72 copy-number losses (HR < 1 and FDR < 25%; e.g., VCP) were found to abrogate the prognostic impact of TLS. These genes need to be further validated. Among the CNAs identified, 206 copy-number gains and 46 copy-number losses led to corresponding gene expression changes (r > 0.3), which might imply their cancer-driving function, and they were, thus, named potential TLS-interacting CNA targets in ovarian cancer (TICTOC) genes (Figure 5B). Among the 206 TICTOC genes with copy-number gains, 14 were found to have known inhibitors (e.g., *DCAF15*) and an additional 48 genes (e.g., STK25) were found to be potentially druggable from the DGIdb database (Figure 5C).

*DCAF15* amplification was one of the top hits to abrogate the prognostic impact of TLS in the TCGA cohort (no. 15; FDR = 23.2%) as well as the HH cohort (HR = 2.27, 95% CI 1.29–72.5, p = 0.0274) (Figure 5B): TLS was associated with overall survival in *DCAF15* wild-type (WT) patients; however, this association was lost in *DCAF15*-amplified patients (Figures 5D and 5E). A consistent trend was observed in the HH cohort, validating the interaction between *DCAF15* and TLS (Figures 5F and 5G). *DCAF15* copy number was frequently increased in over 30% of HGSOC patients. *DCAF15* CNA was significantly correlated with its gene expression, suggesting CNA as an important upstream regulatory mechanism (Figure 5H). DCAF15 forms an E3 ubiquitin-protein ligase complex with CUL4 proteins and mediates protein degradation of substrate proteins, including RBM39, a splicing regulator.^52^^,^^53^ Inhibition of DCAF15 was found to dysregulate the splicing pathway and enhance NK- and T cell-mediated anti-tumor immunity.^53^^,^^54^^,^^55^ We therefore selected *DCAF15* to validate its function to alter anti-tumor immunity.

We examined the gene expression level in a panel of HGSOC cell lines, and DCAF15-amplified cell lines (OVCAR4 and PEO1) showed significantly higher expression of DCAF15 compared with DCAF15-WT cell lines (Figure S6A). Indisulam was previously identified as an aryl sulfonamide anti-cancer drug to dysregulate DCAF15 binding partners and showed strong specificity to DCAF15 and similar functional effect compared with DCAF15 knockout.^52^^,^^53^^,^^54^^,^^56^ Indisulam promotes the interaction between DCAF15 and RBM39 and led to defects in alternative splicing.^53^ We tested the effects of indisulam on OVCAR4 and PEO1 cells and found that RBM39 protein expression was significantly reduced with increased dose of indisulam (Figure S6B).

To understand whether indisulam could help reverse certain mechanisms of TLS function, we co-cultured the two HGSOC cell lines with NK cells, a cell type that was frequently enriched in TLS-rich tumors and phenotypically related to ILC cells (Figures 3G, S3D, and S3J),^57^ using the NK cell line NK-92. Co-culture of NK-92 cells with OVCAR4 and PEO1 significantly reduced cancer cell viability; addition of 125–250 nM indisulam significantly further enhanced the cytotoxic effect of NK-92 cells (OVCAR4, p_NK92:indisulam_ < 0.0001; PEO1, p_NK92:indisulam_ = 0.0232, two-way ANOVA; Figures S6C and S6D). Similarly, the Loewe additivity model showed a strong synergistic effect between NK-92 co-culture and indisulam treatment in both OVCAR4 and PEO1, as an increase in NK-92 cells or indisulam resulted in higher cytotoxicity (darker blue, Figures S6E and S6F) than expected from an additive effect. Consistently, indisulam treatment significantly enhanced caspase-3/7 activation when co-cultured with NK-92 cells (Figures S6G–S6H), inferring increased apoptosis. Granzyme B secretion by NK-92 cells was not significantly affected upon indisulam treatment, suggesting the synergistic effect occurred downstream of NK cell activation (Figure S6I).

The results here suggest that CNAs, including *DCAF15* amplification, may interfere with NK cell-mediated cytotoxicity and thus abrogate TLS function and negatively affect patient outcome.

### Radiomics signature predicts presence of tertiary lymphoid structure in HGSOC

**Figure 6:**
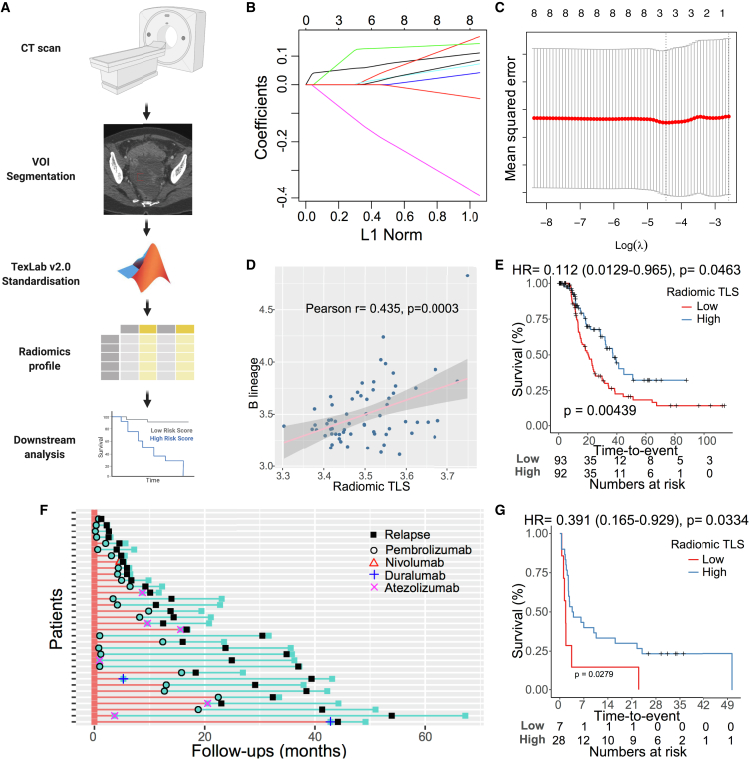
Radiomics signature predicts tertiary lymphoid structure (A) Workflow of radiomics analysis. Tumors in standard-of-care CT images are segmented by an experienced radiologist. The segmented images are normalized and used as input for TexLab 2.0. Radiomics profiles are then used to build the predictive model. (B) The coefficients of radiomics features (y axis) and number of features included in each model (upper x axis) are plotted against shrinkage parameter (lambda). (C) Mean-squared error of each model after 10-fold cross-validation is plotted against lambda in log ratio. (D) Correlation between radiomic TLS score and B cells in the TCGA cohort. Pearson’s correlation coefficient and p value are given. (E) Kaplan-Meier plot of radiomic TLS score associated with progression-free survival in the HH cohort. The p-value is given by log rank test. (F) Summary of patient response to immunotherapy in the HH NSCLC cohort. (G) Kaplan-Meier plot of radiomic TLS score associated with progression-free survival in response to immunotherapy in the HH NSCLC cohort. The p-value is given by log rank test.

We have shown that TLS in tumor is prognostically and functionally important. As TLS is structurally distinct at the histological level, we hypothesized that the presence of TLS in tumors may be detected from computed tomography (CT) scans via quantitative image analysis—radiomics (Figure 6A).

We collected CT scans from 64 and 202 HGSOC patients in the TCGA and HH cohorts, respectively. The 666 radiomics features extracted from segmented and normalized primary tumors were used as input to train a LASSO model to predict TLS in the TCGA cohort^30^ (Figures 6B and 6C). The model was based on three weighted radiomics features: GLCM_Entrop_25HUgl (coefficient 0.109), GLCM_InfCo1_HHL_25HUgl (coefficient 0.0557), and GLCM_sumEnt_HLL_25HUgl (coefficient −0.109). The model output—radiomic TLS score—was significantly correlated with presence of TLS in the TCGA cohort (r = 0.435, p = 0.0003; Figures 6D and S6J). We next computed the radiomic TLS score in the HH cohort and associated it with TLS score measured by CD20 staining and PFS. Radiomic TLS score was significantly correlated with IHC-based TLS score (p = 0.026, Fisher’s exact test; Figure S6K). Consistent with the association between TLS and PFS, radiomic-based TLS score was significantly associated with PFS (HR = 0.112, 95% CI 0.0130–0.965, p = 0.0463; Figure 6E). Moreover, the association between radiomic TLS score and PFS was independent of clinical prognosticators, including stage, age, and post-operative residual disease (HR = 0.0767, 95% CI 0.0086–0.684; p = 0.0214). The performance of radiomic-based TLS score (AUC = 0.574) was similar compared with the IHC-based method (AUC = 0.558) and transcriptomic-based method (AUC = 0.535) (Figure S6L).

The results demonstrated that radiomics-based TLS could be used as a predictor of TLS and survival in HGSOC.

### Radiomics signature predicts response to immunotherapy in NSCLC

TLS was previously shown to predict immunotherapy response in both NSCLC and melanoma patients. Therefore, we aimed to test the potential of radiomics-based TLS in predicting immunotherapy response in NSCLC.

We collected CT images from 26 NSCLC patients in the TCGA cohort and derived a lung cancer-specific radiomic TLS score based on a similar LASSO algorithm in HGSOC. The NSCLC-specific radiomic TLS score was significantly correlated with TLS in the TCGA cohort (r = 0.666, p = 0.0002). The model was then applied to 35 NSCLC patients with CT images available who were treated with immunotherapy from an HH cohort (Figure 6F). Consistent with previous findings, the radiomic TLS score was found to be significantly associated with better PFS after immunotherapy treatment (HR = 0.335, 95% CI 0.140–0.802, p = 0.0141; Figure 6G).

Overall, radiomic TLS score was found to be predictive of TLS and thus, predictive of immunotherapy response in NSCLC.

## Discussion

We comprehensively analyzed the function of TLSs in HGSOC and revealed that TLSs are associated with activated B cell and T cell response against ovarian tumors. TLS was previously identified as both a prognostic marker and a predictive marker of immunotherapy response.^15^^,^^16^^,^^17^ We confirmed the previous findings in both HGSOC and NSCLC using independent cohorts, with two different methods to quantify TLS (i.e., bulk RNA-sequencing estimation and CD20 staining), which highlighted the clinical significance of TLS. We next analyzed the function of TLS using two recent single-cell RNA-sequencing datasets and discovered that TLS-high tumors expressed significantly higher immunoglobulin genes across the IgA and IgG family; for more direct evidence, we showed that TLS-high tumors expressed significantly higher IgA and IgG proteins. Interestingly, a previous study that analyzed an independent single-cell RNA-sequencing dataset found that T cell-high HGSOC tumors contained two distinct B cell populations, plasma cells and plasmablasts, which were responsible for excessive Ig production. Although we did not observe an exclusive presence of the two B cell subtypes within TLS-high tumors, possibly due to intratumoral heterogeneity, we did find that most B cell subtypes in TLS-high tumors expressed higher immunoglobulin genes, highlighting antibody production against the tumor as one function of TLS in HGSOC. Of the IgGs extracted from 98 HGSOC tumors, we observed only three polyclonal IgGs that could recognize HGSOC cell lines. This was consistent with previous findings that patient-derived IgGs often had lower affinity from mixed antigens, including intracellular antigens and cell death antigens from apoptotic cells.^19^^,^^58^ These IgGs may still be clinically relevant since they are expressed within the tumor, and consequently the local concentration is high. In addition, these locally produced IgGs could attract other immune cells such as macrophages and NK cells. Furthermore, some polyclonal IgGs could recognize patient-specific neoantigens, which are present only in the corresponding tumor tissue, instead of cancer cell lines.

Similar to secondary lymphoid structures, TLS as a tumor-localized lymphoid structure could activate both T cells and B cells. Consistent with previous findings, we observed a distinct cytotoxic T cell population (ILCs) enriched in TLS-high tumors compared with TLS-low tumors or tumor-adjacent normal tissues. We also revealed that this cytotoxic T cell population was similar to a recently discovered intratumoral ILTCK population *in vivo*, which was a potent cytotoxic cell type that was activated in early tumors.^42^ This cytotoxic T cell population expressed significantly higher amounts of granzyme B, perforin, and granulysin, which are essential molecules released by cytotoxic T cells during cell-mediated immune response. The evidence presented supports the role of TLSs in proliferation and activation of cytotoxic T cells targeting tumor cells. Interestingly, we observed that TLS-high tumors had significantly higher infiltrating CD8^+^ T cells, but not immune cells from the myeloid lineage, compared with TLS-low tumors. This trend was consistently observed when comparing TLS-high sections with TLS-low sections from different tumors within the same patient. This finding may be explained as follows: (1) tumor-infiltrating CD8^+^ T cells, including ILCs, facilitated TLS development. Indeed, a recent study showed that intratumoral CD8^+^ T cells were essential for TLS development in a mouse model that spontaneously developed TLS.^14^ (2) Perhaps a proportion of the activated cytotoxic T cells, including ILCs, embedded within tumors originated from the TLS instead of from infiltration from secondary lymphoid structures, while the TLS-low tumors contained mostly less cytotoxic T cells, including resident memory T cells. Understanding this conundrum is extremely important because: (1) cytotoxic T cell infiltration is an essential predictor of ICI response.^59^^,^^60^^,^^61^ TLS may be one of the master determinants of T cell infiltration, therefore contributing to its predictive power regarding ICI response. Also, TLS induction may help sensitize TLS-low tumors to ICI.^14^ (2) Chimeric antigen receptor (CAR) T cell therapy, which genetically modifies patients’ autologous T cells to aid recognition of tumor antigens, demonstrates effectiveness in many blood cancer types and less so in solid tumors.^62^^,^^63^^,^^64^^,^^65^ If tumor-associated TLS is responsible for many tumor-specific T cells, inducing and enhancing immunization of TLSs could be an alternative direction for T cell therapy strategies to treat solid tumors.

The formation of TLSs is a multistep process involving immune fibroblast activation and myeloid cell recruitment followed by T and B cell migration.^13^ Among all the cell types involved in TLS formation, CD8^+^ T cells were found to be essential in the process, since depletion of CD8^+^ T cells abrogated formation of the TLS.^14^^,^^66^ Consistent with previous findings, we demonstrated that T cell modulators, *IL15* and *CXCL10*, are often deleted in cancers, and these deletion events coincide with TLS loss in tumors. IL-15 is a cytokine that stimulates proliferation of T lymphocytes and NK cells upon binding with IL-15RA intracellularly, followed by *trans*-presentation to neighboring cells via IL-15RB/IL-2RB and IL-2RG receptors.^67^ It was recently shown that IL-15 was mainly expressed by cancer cells, as well as macrophages in tumors, which is consistent with our findings.^49^ IL-15 was shown to determine both the number and the activity of tumor-infiltrating NK cells and CD8^+^ T cells,^68^ and multiple clinical studies are currently studying the use of IL-15 as an anti-tumor therapy.^69^^,^^70^ Interestingly, recent reports revealed that the generation of an ILTCK population, which was phenotypically similar to ILC and enriched in TLS-high tumors, was highly dependent on IL-15.^42^^,^^43^ IL-15 depletion resulted in impaired ILTCK expansion and differentiation, as well as accelerated tumor growth *in vivo*,^42^^,^^43^ which was consistent with our findings. CXCL10 is a chemokine that is important for T cell recruitment and activation during inflammation.^71^ It is often released by cancer cells undergoing T cell attack, and the expression of CXCL10 was found to be associated with better prognosis in ovarian cancer.^72^ In addition, CXCL10 is reported as a member of a 12-cytokine gene signature to predict TLS and patient survival in colorectal cancer.^73^ Although the deletion of *IL15* and *CXCL10* was consistently observed in independent cohorts of ovarian cancer and lung cancer patients, the direct functional implication of these two genes on TLS formation requires further investigation in future studies. Furthermore, the chromosome region (chr4q35.2) that contains *CXCL10* and *IL15* also contains other known tumor suppressor candidates, including *FAT1*. Therefore, important immune regulators other than CXCL10 and IL15 from chr4q35.2 should also be considered in future functional studies.

Other than CNAs, which could potentially affect TLS formation, we also discovered a list of CNAs (TICTOCs) that could abrogate survival benefits mediated by TLSs. One of the candidate TICTOC genes, *DCAF15*, was found frequently amplified in ovarian cancer, and DCAF15 inhibition led to enhanced NK cell-mediated cytotoxicity, suggesting CNAs as alternative immunotherapy targets in ovarian cancers that are resistant to traditional ICI.^2^^,^^3^^,^^4^ This outcome is consistent with the previous study, which showed DCAF15 in leukemia cells as a negative regulator of NK function.^54^ Mechanistically, DCAF15 inhibition was associated with upregulated expression of the co-stimulatory molecule CD80^54^; DCAF15 inhibition was also shown to disrupt alternative splicing in cancer cells and generates neoantigens, which stimulate T cell-mediated clearance.^55^ Although we did not observe upregulation of CD80 (data not shown) or GZMB secretion upon DCAF15 inhibition, we found a more downstream combination effect between DCAF15 inhibition and NK cells. It suggests: (1) multiple mechanisms may exist in DCAF15-regulated immune responses, and (2) *DCAF15* amplification may blunt immunity delivered by more NK-like, GZMB^+^ cells, such as ILCs. Future studies to functionally validate the proposed TICTOC genes are needed to discover more potential immunotherapy targets in ovarian cancer.

Radiomics extracts quantitative features from standard-of-care CT images, and many potential predictive and prognostic markers have been developed in recent years. Our group previously developed and validated a radiomics prognostic biomarker—RPV—for HGSOC, and RPV was found closely linked to reactive tumor stroma.^30^ In the current study, we used in-house-built radiomics software, TexLab v.2.0, and normalized CT images to develop a radiomic TLS score. We successfully used radiomic profiles to predict TLS in ovarian cancer patients. More importantly, a radiomics-based TLS biomarker predicted response to ICI in NSCLC patients, suggesting the potential of radiomics-based biomarkers in patient stratification for immunotherapies in the future. One pitfall of the study is that the current lung cancer ICI cohort is relatively small, and future studies to validate in a larger clinical trial cohort are required. Moreover, combination of radiomics-based TLS and traditional predictive biomarkers of ICIs such as tumoral PD-L1 expression or TMB may enhance its clinical potential.

To conclude, we define the function of TLS in B cell and T cell maturation to stimulate local immune response against tumors in HGSOC. We discovered CNA could be a general mechanism used by cancer cells to evade TLS-based local immune surveillance. Last, we built a ready-to-use biomarker score to predict TLS and patient survival for clinical translation.

### Limitations of the study

Although we report the clinical importance of TLSs and their associated B cell and T cell response in HGSOC, the direct impact of TLSs on T cell- and B cell-mediated anti-tumor immunity is yet to be functionally characterized. We report that chr4q loss, including IL15 and CXCL10, is associated with the absence of TLS. Nevertheless, genetic and pharmacological inhibition of these candidates is needed to confirm the driver function of chr4q on TLS formation. Since chr4q loss is a broad chromosomal-level loss, multiple immune regulators from chr4q may collectively contribute to TLS formation, and a more comprehensive forward genetic screening approach may be essential. In addition, we report the potential role of DCAF15 in modulating TLS functions; notably, only interactions between NK cells and cancer cells, based on pharmacological inhibition of DCAF15, are investigated. An *in vivo* HGSOC model with high TLS in combination with genetic knockout of *DCAF15* will be needed to further confirm the impact of DCAF15 on TLS function and systematically investigate the phenotypic changes in individual immune cell populations.

## STAR★Methods

### Key resources table

**Table:** 

REAGENT or RESOURCE	SOURCE	IDENTIFIER
**Antibodies**		

CD20	Agilent	M075501-2
CD4	Spring Biosciences	M3352; RRID: AB_11217932
CD8	Spring Biosciences	M5392
Ki67	Agilent	M724029-2; RRID: AB_2687528
CD163	Merck	163M-1; RRID: AB_1159110
DAPI	Thermo	P36935
Human IgA (capture)	Biolegend	411502; RRID: AB_2650697
HRP-conjugated human IgA (detection)	Thermo	PA1-74395; RRID: AB_10985660
Human IgA standard	Thermo	31148
Human IgG (capture)	R&D	MAB11012
HRP-conjugated human IgG (detection)	Biolegend	410902; RRID: AB_2686937
Human IgG standard	R&D	1-001-A
Alexa Fluor 488-conjugated human IgG	Invitrogen	A-11013; RRID: AB_2534080
RBM39	Bethyl laboratories	A300-291A-T
Calnexin	Enzo	adi-spa-860-day
HRP-conjugated rabbit	Dako	P044801-2

**Biological samples**		

Human HGSOC FFPE sections	Imperial College Healthcare Tissue Bank	https://www.imperial.ac.uk/imperial-college-healthcare-tissue-bank/
Human HGSOC fresh frozen DNA samples	Imperial College Healthcare Tissue Bank	https://www.imperial.ac.uk/imperial-college-healthcare-tissue-bank/
Human HGSOC CT scans at diagnosis	Imperial College Healthcare Tissue Bank	https://www.imperial.ac.uk/imperial-college-healthcare-tissue-bank/
Human NSCLC CT scans at diagnosis	Imperial College Healthcare Tissue Bank	https://www.imperial.ac.uk/imperial-college-healthcare-tissue-bank/

**Chemicals, peptides, and recombinant proteins**		

RPMI-1640	Sigma	R5886-500ML
L-glutamine	Thermo	25030081
FCS	First Link	02-05-850
PBS	Internal prepared	NA
Trypsin/EDTA	Sigma	T4049
Myo-inositol	Sigma	I-7508
2-mercaptoethanol	Gibco	21985-023
Folic acid	Sigma	F-8758
Human recombinant IL-2	Peprotech	200-02
Horse serum	ATCC	30-2040
Histo-Clear	National diagnostics	HS-200
Goat serum	Vector Laboratories	S-1000-20
Avidin-biotin complex solution	Vector Laboratories	PK-6100
DAB	Vector Laboratories	SK-4105
DPX	VWR	317616-100ML
Coating buffer	Biolegend	421701
TMB substrates	Biolegend	421101
HRP substrate	Millipore	WBLUF0500
MTT	Sigma	475989

**Critical commercial assays**		

GZMB ELISA assay	R&D	DY2906-05
Caspase 3/7 assay	Merck Millipore	MCH100108
BCA assay	Thermo	23225

**Deposited data**		

Whole exome sequencing for HGSOC	This study	Mendeley Data: https://doi.org/10.17632/h98925j75g.21
Bulk RNA-sequencing data for HGSOC	TCGA	https://xena.ucsc.edu/
Bulk RNA-sequencing data for NSCLC	TCGA	https://xena.ucsc.edu/
CNA data for HGSOC	TCGA	https://xena.ucsc.edu/
CNA data for NSCLC	TCGA	https://xena.ucsc.edu/
Single cell RNA-sequencing data	Qian et al.,^39^ Olbrecht et al.^46^	https://lambrechtslab.sites.vib.be/en
Single cell RNA-sequencing data	Olalekan et al.^41^	GEO: GSE14708244
Single cell RNA-sequencing data	Davies et al.^74^	GEO: GSE165897

**Experimental models: Cell lines**		

PEO1	ECACC	10032308
OVCAR4	MERCK	SCC258
OSEC	Zhang et al.^75^	Gift
OVCAR3	ATCC	HTB-161
NK-92	ATCC	CRL-2407

**Software and algorithms**		

MCP counter	Becht et al.^38^	https://github.com/ebecht/MCPcounter
Seurat 4.3.0	Pinato et al.^76^	https://satijalab.org/seurat/
SingleR	Birtley et al.^77^	https://github.com/dviraran/SingleR
ggplot2	Aran et al.^78^	https://github.com/tidyverse/ggplot2
Glmnet	Li et al.^79^	https://cran.r-project.org/web/packages/glmnet/index.html
survival	Li et al.^80^	https://cran.r-project.org/web/packages/survival/index.html
Limma	Talevich et al.^81^	http://bioconductor.org/packages/release/bioc/html/limma.html
BWA	Carter et al.^82^	https://github.com/lh3/bwa
samtools	Mermel et al.^83^	https://samtools.sourceforge.net/
PicardTools	Broad institute	https://broadinstitute.github.io/picard/
CNVkit	Arshad et al.^84^	https://cnvkit.readthedocs.io/en/stable/
ABSOLUTE	Hao et al.^85^	https://github.com/genepattern/genepattern-server/releases/tag/v3.9_22.11_b389
GISTIC2.0	Wickham et al.^86^	https://cloud.genepattern.org
TexLab v2.0	Lu et al.^30^	Available upon request
R 4.1.0	The R Project for Statistical Computing	https://www.r-project.org
GraphPad PRISM 6	GraphPad software	https://www.graphpad.com/
BioRender	BioRender website	https://www.biorender.com/

### Resource availability

#### Lead contact

Further requests for information and materials will be fulfilled by the lead contact, Prof Eric Aboagye (eric.aboagye@imperial.ac.uk).

#### Materials availability

All unique/stable reagents generated in this study are available from the lead contact with a completed Materials Transfer Agreement.

### Experimental model and subject details

#### Patients

The current study was complied with the ethical standards of the institutional and/or national research committee and the principles of the 1964 Declaration of Helsinki and its later amendments. Ethical approval for retrospective analysis of human bio-specimen was obtained under the Imperial College Healthcare Tissue Bank Review Committee (ICHTB HTA licence: 12275; REC Wales approval: 17/WA/0161).

HGSOC and NSCLC patients from Hammersmith cohort were included in this study from 2004-2018, depending on the pre-operative CT scan availability. For the HGSOC cohort, female patients with age at diagnosis between 30-91 were included; for the NSCLC cohort, 21 male patients and 16 female patients with age at diagnosis between 38-80 were included. All patients were treated at Imperial College Healthcare NHS trust. For HGSOC bio-specimen collection, additional criteria including pre-operative, pre-chemotherapy and tumour cellularity were applied. Tumour cellularity was determined by an experienced pathologist (S.D.) using hematoxylin and eosin-stained sections. Only tumour bio-specimen with over 50% tumour cellularity were included in the whole exome sequencing analysis. Sample size of the patient cohort was calculated based on previous studies^30^ (alpha=0.5, beta=0.2, q1(exposed)=0.3, relative hazard=0.6), which led a total of 230 cases needed for the current study. Patients were allocated to the experimental groups based on the presence of tertiary lymphoid structures in the primary tumours and availability of pre-operative CT scans.

#### Cell culture

NK-92, OVCAR4, PEO1 and OVCAR3 cell lines were purchased from American Type Culture Collection (ATCC). The OSEC cell line was a gift from Michael J. O’Hare.^74^ The OSEC cell line was generated from immortalisation of ovarian surface epithelium cells with hTERT and a temperature sensitive form of SV40 Large T antigen. The OSEC, OVCAR4, PEO1 and OVCAR3 cells were cultured in RPMI1640 medium (Sigma) supplemented with 10% FCS (FirstLink) and 2 mM L-glutamine (LifeTechnologies). The OSEC cells were cultured at 33°C whereas the other cells were cultured at 37°C. Adherent cell lines were passaged at 70% confluency by washing with PBS, followed by detaching with trypsin/EDTA solution.

NK-92 is an IL-2 dependent natural killer cell line derived from peripheral blood mononuclear cells of a patient with non-Hodgkin's lymphoma. NK-92 cells were cultured in Alpha MEM supplemented with 0.2mM myo-inositol, 0.1mM 2-mercaptoethanol, 0.02 mM folic acid, 100 IU/mL human recombinant IL-2, 12.5% FCS and 12.5% horse serum. All cell lines underwent weekly mycoplasma testing.

OVCAR3, OVCAR4 and PEO1 were all originally derived from HGSOC tumours, and characterised by TP53 mutations and extensive genomic arrangements, which closely resemble the HGSOC patient cohort analysed (TCGA and HH cohort). OVCAR4 and PEO1 carry DCAF15 amplifications (19p13), whereas OVCAR3 carry CCNE1 amplification (19q12).

#### Public datasets

HGSOC and NSCLC cases from the Cancer Genome Atlas project were used as validation cohorts in this study. The bulk RNA-sequencing (pan-cancer normalised) and gene-level copy number alterations (GISTIC2 estimate) data for both HGSOC and NSCLC cohorts were downloaded from UCSC Xena (https://xena.ucsc.edu/).

Two single cell RNA-sequencing datasets from 9 HGSOC patients were downloaded from https://lambrechtslab.sites.vib.be/en.^39^^,^^46^ Additional two single cell RNA-sequencing datasets (GSE147082^41^ and GSE165897^75^) were downloaded from GEO (https://www.ncbi.nlm.nih.gov/geo/).

### Method details

#### Immunohistochemistry

Sections were deparaffinised in two changes of Histo-Clear (National Diagnostics USA) and then rehydrated using graded alcohols (100, 90, and 70%). Sections were washed in running water before proceeding for blocking endogenous peroxidase activity using 0.3% hydrogen peroxide (Sigma-Aldrich Ltd) in PBS, followed by several washes in distilled water. Pre-treatment was carried out by boiling slides in Citrate buffer (pH 6) at highest setting in a microwave. Sections were rinsed in distilled water then blocked using 1.5% normal goat serum (Vector Laboratories) at room temperature (RT) to reduce non-specific binding, followed by overnight incubation in the primary antibody (CD20, CD163 and Ki67). After washes in PBS, the sections were incubated with a secondary antibody (Vector Laboratories) for 30 min at RT. Sections were washed in PBS and incubated in the avidin-biotin complex solution (VECTASTAIN Elite ABC Kit, Vector Laboratories) at RT. After washes in PBS, chromogenic reaction was developed using DAB (Diaminobenzidine, Vector ImmPACT DAB Peroxidase Substrate). The DAB solution was washed off and the tissues were counterstained in Mayer’s Haematoxylin (Sigma Aldrich). The tissue sections were washed in running water and then dehydrated in graded alcohol (70%, 90%, and absolute alcohol), cleared in two changes of Histo-Clear, and then mounted with DPX (VWR BDH ProLab) mounting medium.

#### Multiplexed IHC

CD4, CD8, FOXP3 and PD-1 was stained on 2μm FFPE sections from human HGSOC using a previously optimized method.^76^ CD8^+^, CD4+/FOXP3 and CD8+/PD-1+ co-stained cells were assessed at 450x magnification across the entire section including both cancerous and non-cancerous area and reported as cellular density per mm^2^ of tissue. Each section was scored based on average of three independent regions. The score was assessed and agreed by two independent pathologists (D.P. and F.M.).

#### ELISA

ELISA was performed. Approximately 2mm^2^ ovarian tumour tissues were homogenised in 1mL RIPA buffer (Sigma) using Precellys tissue homogenizer (Bertin Instruments). The protein concentration was determined by BCA assay (Thermo). 96-well plate was coated with 0.25ug/mL anti-human IgA (Biolegend, 411502), 0.25ug/mL anti-human IgG antibody (R&D, MAB11012), or 0.8ug/mL anti-human GZMB antibody (R&D, DY2906-05) diluted in coating buffer (Biolegend, 421701). The coated plate was then blocked with 1X Assay Diluent A (Biolegend) and washed with 0.05% PBST. Standard samples (human IgG: R&D, 1-001-A; human IgA: Thermo, 31148; human GZMB: R&D, DY2906-05) and tissue lysate was diluted in Assay Diluent A and loaded onto the plate. After 2h incubation at room temperature, the plate is washed with 0.05% PBST. The plate was then incubated with detection antibody (HRP conjugated anti-IgG: Biolegend, 410902; HRP conjugated anti-IgA: Thermo, PA1-74395; anti-GZMB: R&D, DY2906-05) and subsequently TMB substrates (Biolegend, 421101). After 15min incubation at room temperature, stop solution (0.16M H_2_SO_4_) was added and the absorbance was read at 450nm.

#### Immunofluorescent microscopy

The immunofluorescent microscopy was performed using methods previously described.^77^ Briefly, 1 × 10^5^ ovarian cancer cells (OVCAR3, OVCAR4 and PEO1) and negative control cells (OSEC) were seeded on coverslips and incubated overnight. The cells on coverslips were fixed in 4% PFA and incubated overnight with IgG antibodies extracted from ovarian tumour tissues diluted 1:100 in PBS and 1% FCS. The coverslips were then incubated with 1:200 anti-human IgG (Invitrogen, A-11013) and mounted with DAPI (Invitrogen, P36935). The images were taken using confocal microscope (Leica SP5). The confocal images were analysed using Fiji.

#### NK co-culture

On day 0, 5000 cancer cells (OVCAR4 or PEO1) were seeded on 96-well plate in triplicates. On the next day, 0, 2500 or 3750 NK-92 cells were added to cancer cells, followed by addition of 0-250nM indisulam (Sigma, SML1225). The culturing medium for NK and medium for cancer or non-malignant cell lines were mixed at 1:1 ratio. After 2 days incubation, NK-92 cell-containing media were removed and cells were washed 3 times with PBS. The cells in the plate were read by MTT assay (Sigma, M5655) following manufacturer’s protocol.

For caspase3/7 assay, cancer cells were incubated with NK-92 cells in 1:0.75 ratio and DMSO or 250nM indisulam for 1 day, followed by washing with PBS. The cancer cells were detached from plates and incubated with Muse Caspase-3/7 Kit (Merck Millipore, MCH100108) following manufacturer’s protocol. The percentage of apoptotic cells were analysed using Muse cell analyzer.

#### Immunoblotting

5 × 10^5^ OVCAR4 or PEO1 cells were seeded on 6-well plate and treated with 0-250nM indisulam on the day 1. Cells were harvested with RIPA buffer supplemented with protease inhibitor on day 2. Protein lysate was quantified using BCA assay, and 20ug proteins were loaded for SDS-PAGE electrophoresis followed by western blot (Bio-rad). Proteins in membrane were incubated with primary antibodies including anti-RMB39 (1:1000, Bethyl laboratories, A300-291A-T) and anti-Calnexin (1:10,000, Enzo, adi-spa-860-d), followed by HRP-conjugated secondary antibodies including anti-rabbit (Dako, P044801-2). Upon incubation with HRP substrate (Millipore, WBLUF0500), protein bands were visualised by autoradiography.

### Quantification and statistical analysis

#### Single-cell RNA sequencing analysis

The count matrix and metadata were downloaded and read as a Seurat object and analysed as following steps: 1) The expression matrix was filtered for less than 6000 RNA features and less than 15% mitochondria genes. 2) The expression matrix was log normalised with scale factor of 10000, and 3) ‘vst’ method was used to find 3000 most variable features. 4) The expression matrix was then scaled with RNA count, mitochondria percentage, cell cycle scores as variables to regress out. 5) Principal component analysis was performed, followed by clustering with optimised dimensions and resolutions. 6) The clusters were visualised using UMAP with optimised dimensions. 7) The cell clusters were labelled using ‘singleR’ and ‘celldex’ package,^78^ and manually curated with top expressed marker genes. Each cell cluster was sub-grouped using the step (1 - 7).

#### Whole exome sequencing

Total DNA were extracted from ovarian tumour tissues using Qiagen DNeasy blood and tissue kit following manufacturer’s protocol. The DNA then underwent quality control using Agilent Bioanalyzer. The whole exome was captured using Agilent SureSelect target enrichment system, followed by loading onto BGI DNBseq platform. Briefly, qualified DNA were firstly fragmented into 150-200bp and adapters were added; The adapter-DNA were purified and amplified by ligation-mediated polymerase chain reaction, followed by hybridisation to SureSelect Biotinylated RNA Library (BAITS) for enrichment; Hybridized fragments were bound to streptavidin beads and captured products were qualified using Agilent 2100 Bioanalyzer. The qualified exome library was loaded onto BGI DNBseq-500 platform for high-throughput sequencing until desired sequencing coverage was met.

#### Copy number calling

The raw sequencing reads in fastq format were aligned to GRCh38 (release 98) genome using the Burrows-Wheeler Aligner (BWA).^79^ The resulting sam file was converted into bam and sorted using samtools.^80^ The duplicate reads were removed using ‘MarkDuplicates’ function from PicardTools.

The bam file filtered for duplicates were used as input for CNVkit^81^ to infer copy number profile from sequencing data. 5kb was set as the short regions to be overlapped by neighbouring regions when calculating the sequencing accessibility of the GRCh38 genome. All non-malignant samples were pooled to make the normal reference. All the tumour samples were batch processed and output as copy number segments file. To remove tumour sample purity and ploidy problem, ABSOLUTE^82^ was applied and absolute cellular copy number of DNA segments were given as a result. The copy number frequency plot was generated using ‘cnFreq’ from ‘GenVisR’ package. GISTIC 2.0^83^ was applied to identify the significantly recurrent chromosome regions using GenePattern (https://cloud.genepattern.org/) in both TCGA and HH cohort (amplifications threshold=0.1, deletions threshold =0.1, confidence level=0.9, q value threshold =0.25, focal length cutoff=0.8).

#### Mutation calling

The bam file filtered for duplicates were also used to identify mutations (both SNP and indels) from sequencing data. Firstly, pileup files were generated using mpileup function from Samtools. VarScan v2.3.7 was then used to identify SNPs and indels using ‘mpileup2snp’ and ‘mpileup2indel’, respectively. The variant calling file was then annotated with SnpSift and common variants were removed. The variant effect was also annotated using ‘variant_effect_predictor’ and non-synonymous variants were kept.

#### Radiomics analysis

Pre-operative CT images were downloaded from Imperial College London NHS trust or the Cancer Imaging Archive (TCIA; http://www.cancerimagingarchive.net/). Primary ovarian tumour masses on CT scans were segmented and checked using ITK snap v3.2, by four experienced radiologists (G.W., M.C., A.R. and S.C.). Both cystic and solid parts of the primary tumour were included in the segmentation. Both primary masses in bilateral tumour cases were segmented and radiomics profile was separately generated for each mass.

The segmented images were resampled to standard slice thickness of 1mm X 1mm X 3mm before used as input to in-house developed Texture analysis software- TexLab v2.0.^84^ The software generated 666 radiomic features including 1. Shape and Size features; 2. First-order statistics; 3. Second-order statistics; 4. Wavelet features.^30^

#### TLS-RPV development

To develop the TLS-RPV model in ovarian cancer, the radiomic matrix from TCGA HGSOC cohort was firstly scaled by mean and centered. The same scaling and centering factor was applied to HH cohort. All radiomic features that weakly correlated (Spearman correlatin >0.1 or < -0.1) with TLS marker in the TCGA HGSOC cohort were included as input for LASSO. 10-fold cross-validation was then performed to regress radiomic feature combination with TLS marker using ‘cv.glmnet’ function. The radiomic feature combination which produced the minimal error was selected as the final model. The model was used to generate TLS-RPV for each ovarian cancer case from HH HGSOC cohort. For bilateral cases, the tumour mass which produced higher TLS-RPV value was selected to represent the case.

A similar pipeline was adapted to develop TLS-RPV in NSCLC cohort with a few modifications. Firstly, standardised radiomics features correlated with TLS marker (Spearman correlation >0.3 or < -0.3) in TCGA lung cancer cohort were included as LASSO input. Secondly, 3-fold cross-validation was applied instead due to lower sample size. The model was then applied to radiomic profile from HH NSCLC cohort.

#### Statistical analysis

All the bioinformatics analyses were performed using R 4.1.0. The R packages used in this study includes ‘MCP counter’,^38^‘Seurat 4.3.0’,^85^ ‘ggplot2’,^86^ ‘glmnet’^87^ and ‘survival’.^88^ The Kaplan-Meier plot was generated using ‘ggkm’ package and log-rank test was used to test the survival difference. Differential gene expression/CNA enrichment was performed using ‘limma’^89^ package. The viability assays were analysed using Graphpad Prism 6. The interaction between indisulam and NK-92 was analysed using 2-way ANOVA test. 2-sided unpaired t-test was used to compare two sample groups. Illustrative diagram was generated using BioRender.

## Data Availability

• Single-cell RNA-sequencing data used in this study are available at GEO and accession numbers are listed in the key resources table. Copy number data and clinical data have been deposited at Mendeley (https://doi.org/10.17632/h98925j75g.1) and are publicly available as of the date of publication. The DOI is listed in the key resources table. Raw sequencing, CT imaging data, and microscopy data reported in this paper will be shared by the lead contact upon reasonable request. • No original code is reported in this study. • Any additional information required to reanalyze the data reported in this paper is available from the lead contact upon request.

## Supplemental information

Document S1. Figures S1–S6

Table S1. Altered copy-number regions associated with presence of TLS in the TCGA ovarian cancer cohort, related to Figure 4

Table S2. Multivariable Cox regression results of gene copy-number alterations interacting with TLS in the TCGA ovarian cancer cohort, related to Figure 5

Document S2. Article plus supplemental information
