# Supplementary material for: Tumor and local lymphoid tissue interaction determines prognosis in high-grade serous ovarian cancer
Source: Cell Rep Med. 2023 Jun 21;4(7):101092. doi: 10.1016/j.xcrm.2023.101092 (PMC10394173; doi:10.1016/j.xcrm.2023.101092)
Supplement: Document S1. Figures S1–S6 [file mmc1.pdf]

Cell Reports Medicine, Volume *4*

Supplemental information

Tumor and local lymphoid tissue interaction determines prognosis in high-grade serous ovarian cancer

Haonan Lu, Hantao Lou, Georg Wengert, Reema Paudel, Naina Patel, Saral Desai, Bill Crum, Kristofer Linton-Reid, Mitchell Chen, Dongyang Li, Jacey Ip, Francesco Mauri, David J. Pinato, Andrea Rockall, Susan J. Copley, Sadaf Ghaem-Maghami, and Eric O. Aboagye

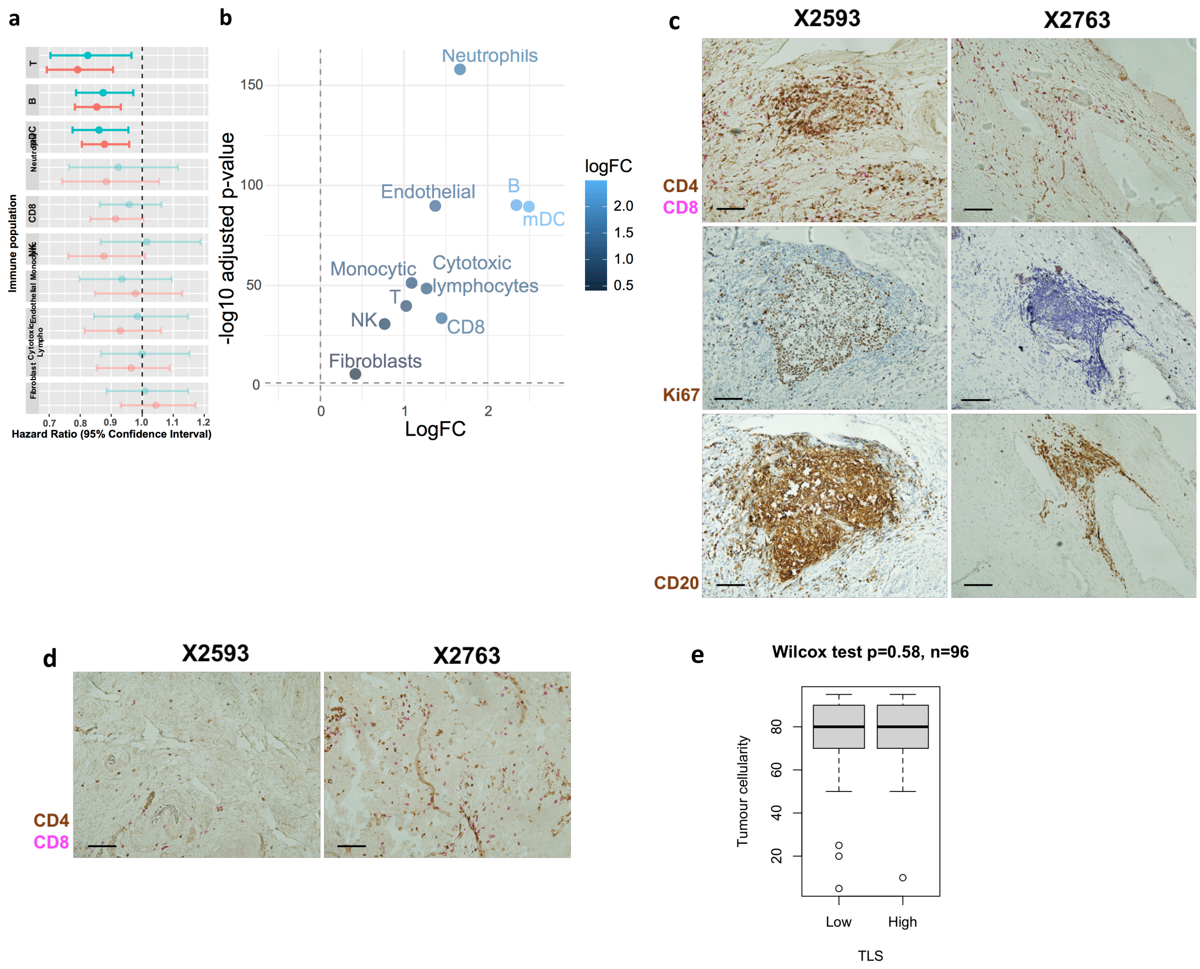

Supplementary Figure 1. Immune signature in lung cancer and HGSOC. a Summary of prognostic potential of immune cell subpopulations in TCGA lung adenocarcinoma cohort. n=567. **b** Volcano plot comparing immune cell subpopulation between lung and ovarian tumours. **c** Representative IHC images stained for Ki67 (top panel), CD4 and CD8 (middle panel), and CD20 (lower panel) and from two HGSOC cases (X2593 and X2763). All three panels are from sequential FFPE sections, indicating the co-localisation of Ki67, CD4, CD8, and CD20. **d** Representative IHC images stained for CD4 and CD8 from two HGSOC cases (X2593 and X2763), indicating the infiltrating T cells outside TLS. Scale bar indicates 100µm. The images are taken from the same sections as middle panel in **c**. **e** Boxplot showing the association between tumour content with TLS from HH cohort. P-value is given by two-sided t-test. Related to Figure 1.

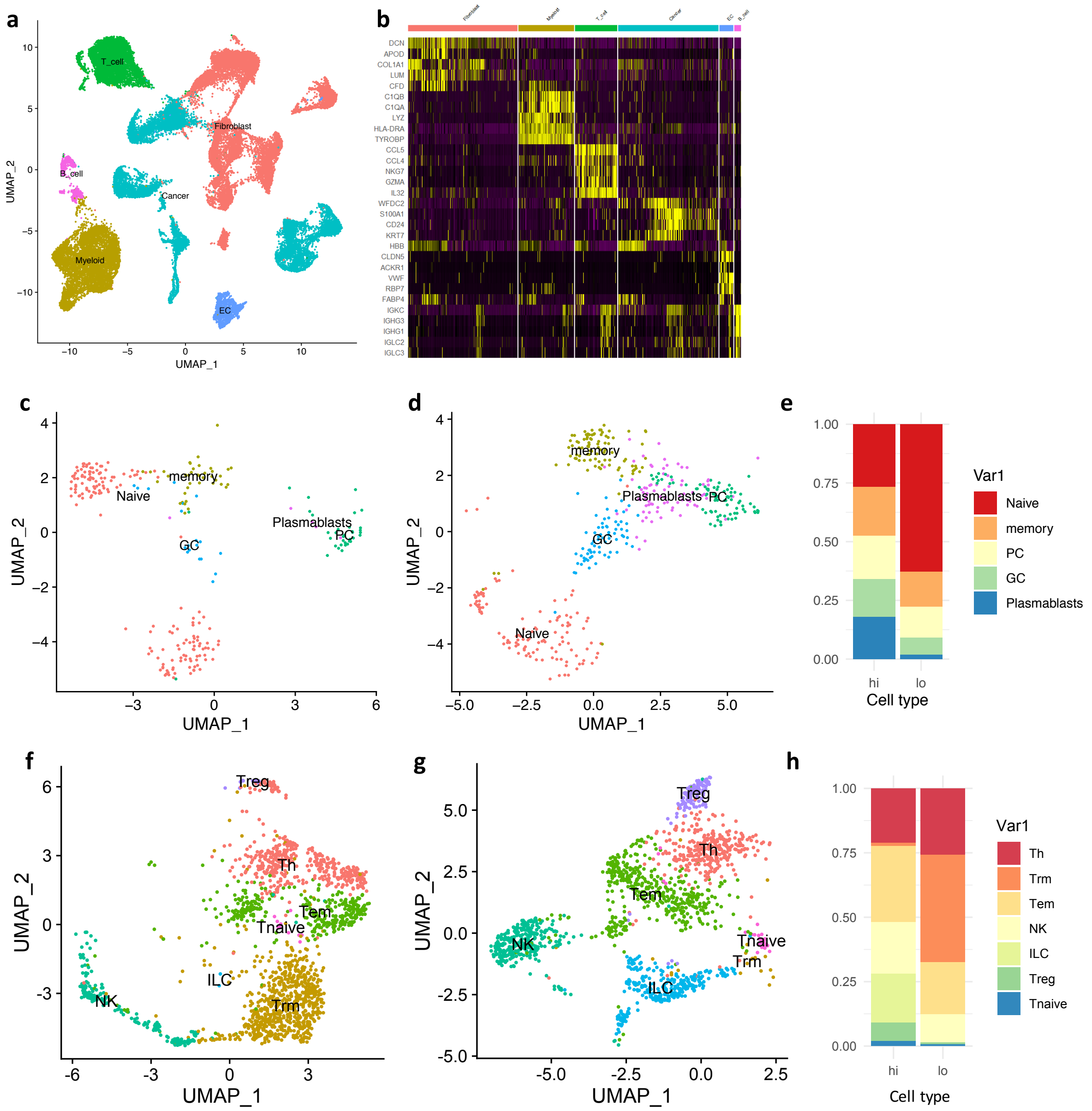

Supplementary Figure 2. Cell subtype clustering in HGSOC RNA-sequencing dataset (Lambrechts cohort) comparing TLS-low (n=3; 18,354 cells) with TLS-high tumours (n=2; 5,996 cells). **a** UMAP analysis of 24,350 single cells revealed cell clusters representing 6 unique cell types. **b** The expression of top marker genes in the 6 cell subtypes. UMAP analysis of B cell subtypes in **c** TLS-low and **d** TLS-high tumours. **e** Bar plot showing the proportion of B cell subtypes comparing TLS-low with TLS-high tumours. UMAP analysis of T cell subtypes in **f** TLS-low and **g** TLS-high tumours. **h** Bar plot showing the proportion of seven T cell subtypes comparing TLS-low with TLS-high tumours. Related to Figure 2 and 3.

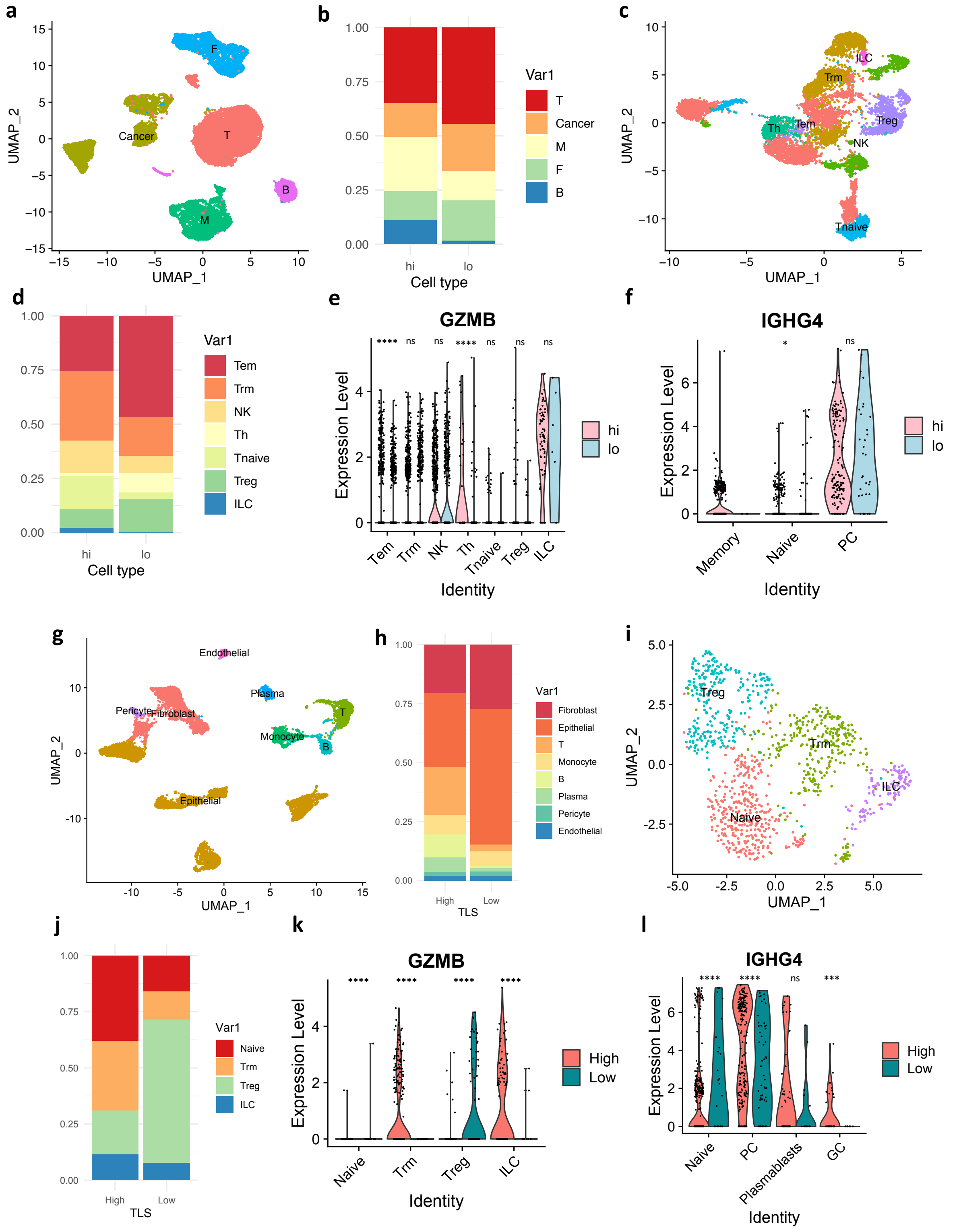

Supplementary Figure 3. Characteristics of T-cell and B-cell subtype associated with TLS in a-f GSE165897 and g-l GSE147082. **a** UMAP analysis showing the cell clusters in GSE165897. F, fibroblasts; B, B-cells; T, T-cells; M, myeloid cells. **b** Proportion of cell clusters associated with TLS status. TLS high: n=5, 9368 cells; TLS low: n=6, 11854 cells. **c** UMAP analysis of T cell subclusters. Tem, effector memory T-cells; Trm, resident memory T-cells; Th, T helper cells; Tnaive, Naïve T-cells; Treg, regulatory T-cells; ILC, innate lymphoid cells. **d** Proportion of T-cell subtypes associated with TLS. **e** *GZMB* expression across T-cell subtypes. **f** *IGHG4* expression across B-cell subtypes. **g** UMAP analysis showing the cell clusters in GSE147082. B, B-cells; T, T-cells. **h** Proportion of cell clusters associated with TLS status. TLS high: n=2, 4602 cells; TLS low: n=4, 5132 cells. **i** UMAP analysis of T cell subclusters. Trm, resident memory T-cells;

naive, Naïve T-cells; Treg, regulatory T-cells; ILC, innate lymphoid cells. **j** Proportion of T-cell subtypes associated with TLS. **k** *GZMB* expression across T-cell subtypes. **l** *IGHG4* expression across B-cell subtypes. P-values are given by Wilcoxon Rank Sum test. Related to Figure 2 and 3.

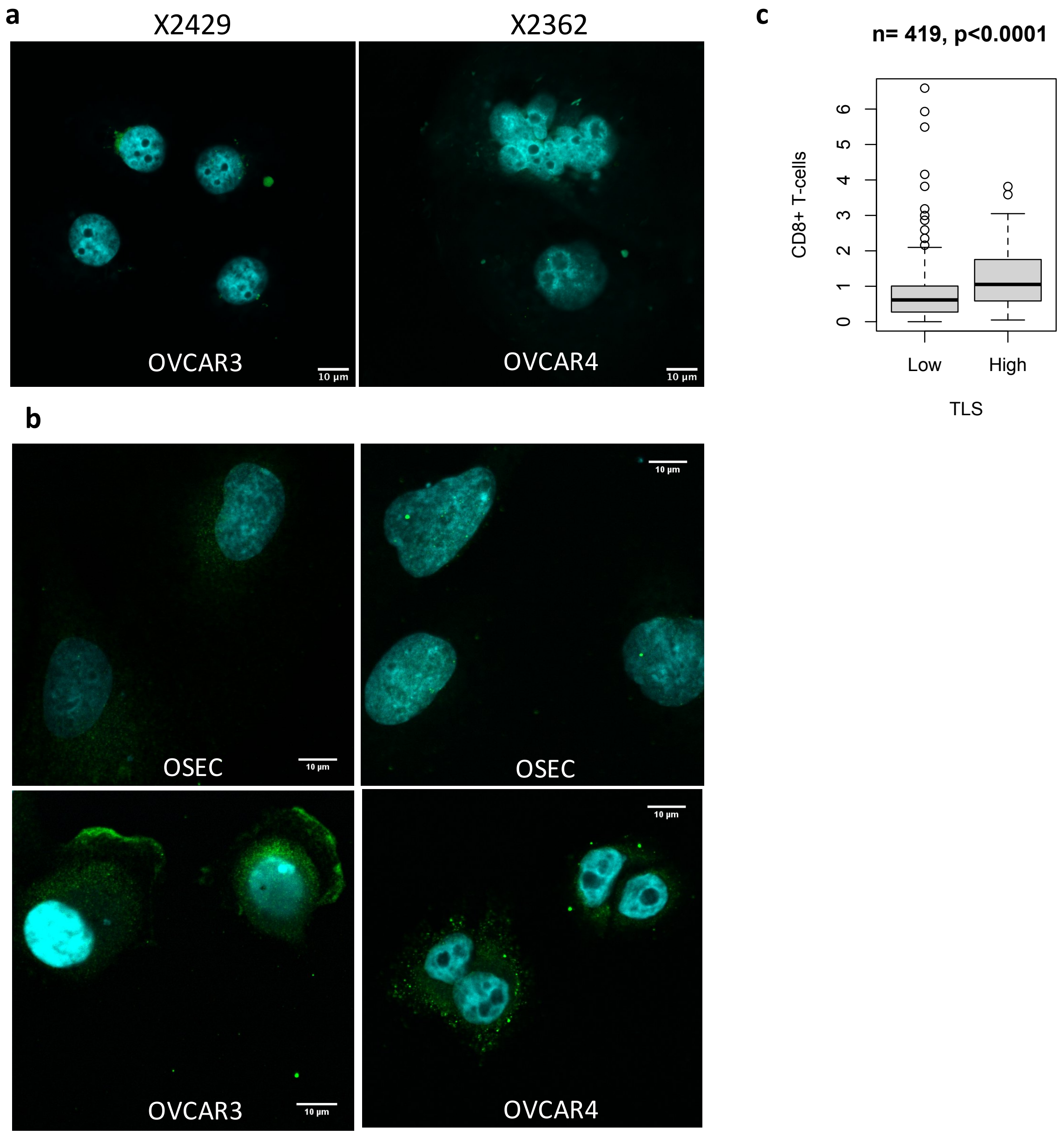

Supplementary Figure 4. a Immunofluorescence image showing OVCAR3 and OVCAR4 cells stained with human IgG control antibody. **b** Immunofluorescence microscopy images of OSEC (upper panel), OVCAR3 (lower left) and OVCAR4 (lower right) stained by polyclonal IgGs extracted from two HGSOC tumours (X2429 and X2362; 1.5ng/ul). Cyan, DAPI; green, human IgG. Scale bar represents 10 micron **c** CD8+ T-cell level in TLS-low compared to TLS-high ovarian tumours in the TCGA cohort. CD8+ T-cell level is estimated from bulk-RNA-sequencing data using MCP-counter. TLS high: n=314; TLS low: n=314. P-value is given by 2-sided t-test. Related to Figure 2 and 3.

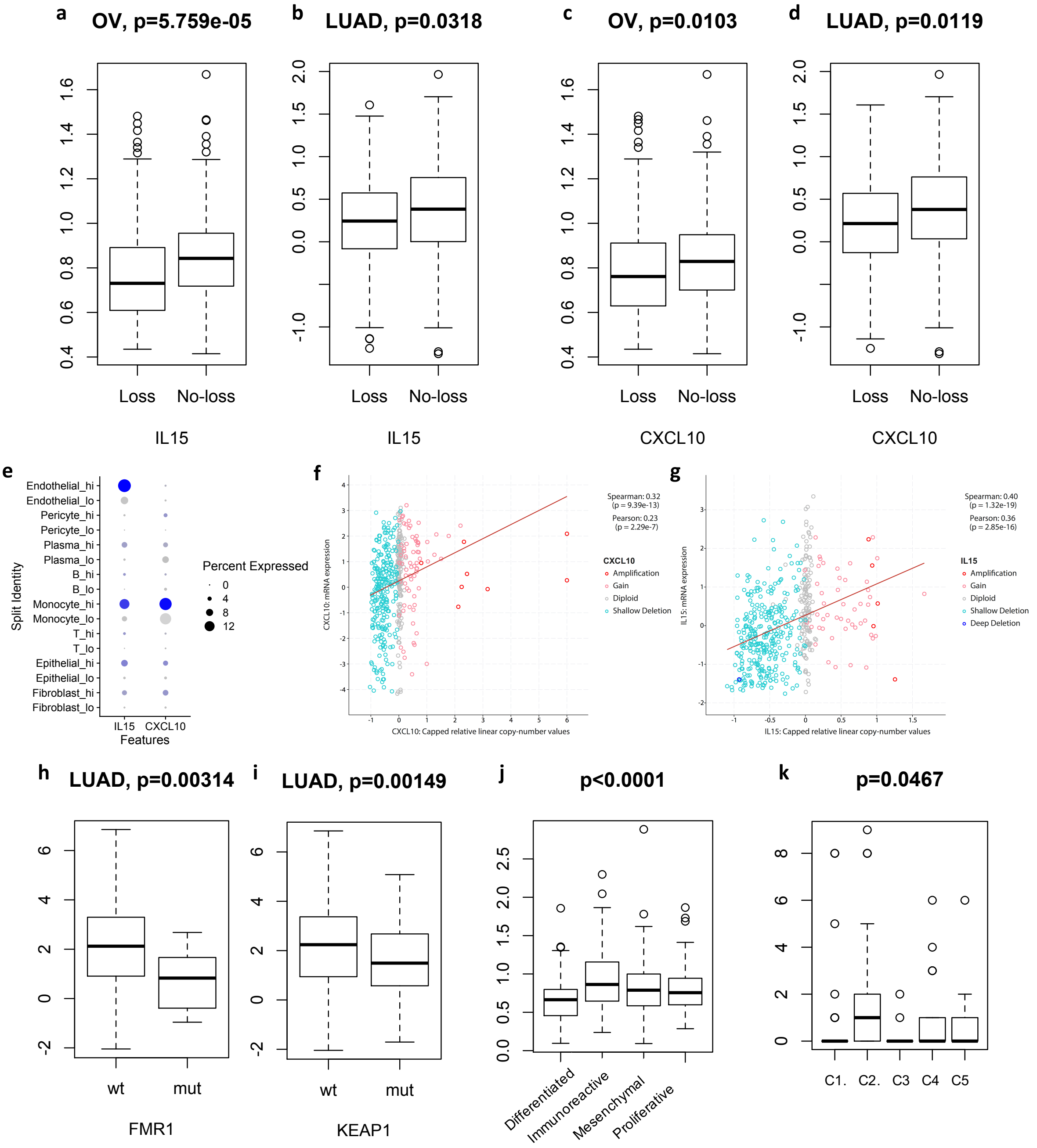

Supplementary Figure 5. Genomic characteristics associated with TLS. The association between TLS and copy number alterations of **a,b** *IL15* and **c,d** *CXCL10* in **a,c** ovarian cancer (n= 407) and **b,d** lung adenocarcinoma (n= 512) from the TCGA cohort. y-axis, TLS level estimated by presence of B-cells. **e** IL15 and CXCL10 expression across cell types comparing TLS-high with TLS-low tumours in GSE147082 dataset. The correlation between copy number status and mRNA expression level for **f** *IL15* and **g** *CXCL10* in TCGA cohort. The Spearman and Pearson correlation coefficients are given. The association between TLS and mutational status of **h** *FMR1* and **i** *KEAP1* in lung adenocarcinoma from TCGA cohort (n= 509). P-values are given by 2-sided t-test. y-axis, TLS level estimated by presence of B-cells. The association between TLS and molecular subtypes in ovarian cancer from **j** TCGA (n= 411) and **k** HH cohort (n=112). P-values are given by Kruskal-Wallis test. y-axis, TLS level estimated by presence of B-cells. Related to Figure 4.

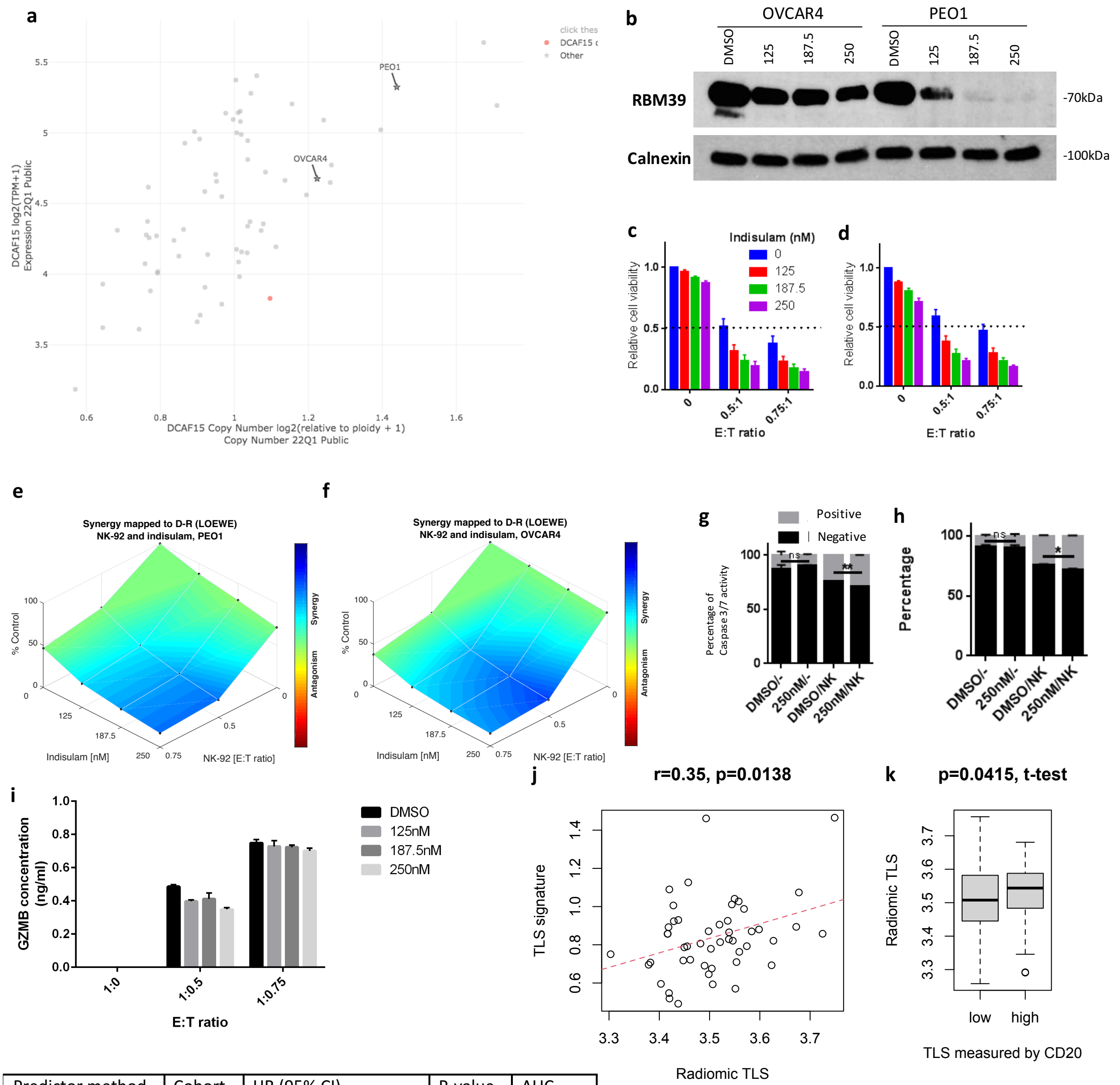

l

Predictor method	Cohort	HR (95% CI)	P-value	AUC
Radiomic TLS	HH	0.112 (0.0129-0.965)	0.0463	0.574
IHC of B cells	HH	0.542 (0.322-0.913)	0.0213	0.558
Transcriptional signature	TCGA	0.661 (0.478-0.914)	0.0124	0.535

Supplementary Figure 6. The role of DCAF15 in regulating TLS function. a The correlation between expression and copy number status of DCAF15 in ovarian cancer cell lines. **b** Immunoblotting of RBM39 when OVCAR4 and PEO1 are treated with increasing dose of indisulam. Calnexin is used as the internal control. Cell viability when **c** PEO1 and **d** OVCAR4 are co-cultured with NK-92 cells and increasing dose of indisulam. n=5. The synergy map between NK-92 and indisulam in **e** PEO1 and **f** OVCAR4 at increasing dose of NK-92 cells (bottom right axis) and indisulam (bottom left axis). Blue color indicates presence of synergy whereas red color indicates antagonism. Caspase3/7 assay in **g** PEO1 and **h** OVCAR4 cells treated with and without 250nM indisulam and NK-92 cells in E:T ratio of 1:0.75. n=5. **i** The expression of GZMB in OVCAR4 cells co-cultured with NK-92 and indisulam is measured by ELISA. ****, p<0.0001; ***, p<0.001; **, p<0.01; *, p<0.05; ns, p>0.05. The significance is given by 2-sided paired t-test. Data are presented as mean values ± SEM. **j** Scatter plot showing the correlation between radiomics-based TLS and transcriptome-based TLS signature from the TCGA cohort. Correlation coefficient and p-value is given by Pearson's correlation. n=48. **k** Association between radiomics-based TLS and IHC-based TLS status from HH cohort. P-value is given by two-sided t-test. TLS high: n=70; TLS low: n=193. **l** Comparison of radiomics-based TLS with IHC-based and transcriptomics-based method. Hazard ratio (HR), p-value and area under curve (AUC) are given by Cox-regression analysis. Related to Figure 5 and 6.
